# Changes in walking function and neural control following pelvic cancer surgery with reconstruction

**DOI:** 10.3389/fbioe.2024.1389031

**Published:** 2024-05-17

**Authors:** Geng Li, Di Ao, Marleny M. Vega, Payam Zandiyeh, Shuo-Hsiu Chang, Alexander. N. Penny, Valerae O. Lewis, Benjamin J. Fregly

**Affiliations:** ^1^ Rice Computational Neuromechanics Laboratory, Department of Mechanical Engineering, Rice University, Houston, TX, United States; ^2^ Biomotion Laboratory, Department of Orthopedic Surgery, McGovern Medical School at the University of Texas Health Science Center at Houston, Houston, TX, United States; ^3^ Department of Physical Medicine and Rehabilitation, McGovern Medical School at the University of Texas Health Science Center at Houston, Houston, TX, United States; ^4^ Department of Orthopedic Oncology, University of Texas MD Anderson Cancer Center, Houston, TX, United States

**Keywords:** pelvic sarcoma, custom implant, orthopedic oncology, instrumented gait analysis, walking biomechanics, muscle synergies, neuromusculoskeletal modeling

## Abstract

**Introduction:** Surgical planning and custom prosthesis design for pelvic cancer patients are challenging due to the unique clinical characteristics of each patient and the significant amount of pelvic bone and hip musculature often removed. Limb-sparing internal hemipelvectomy surgery with custom prosthesis reconstruction has become a viable option for this patient population. However, little is known about how post-surgery walking function and neural control change from pre-surgery conditions.

**Methods:** This case study combined comprehensive walking data (video motion capture, ground reaction, and electromyography) with personalized neuromusculoskeletal computer models to provide a thorough assessment of pre- to post-surgery changes in walking function (ground reactions, joint motions, and joint moments) and neural control (muscle synergies) for a single pelvic sarcoma patient who received internal hemipelvectomy surgery with custom prosthesis reconstruction. Pre- and post-surgery walking function and neural control were quantified using pre- and post-surgery neuromusculoskeletal models, respectively, whose pelvic anatomy, joint functional axes, muscle-tendon properties, and muscle synergy controls were personalized using the participant’s pre-and post-surgery walking and imaging data. For the post-surgery model, virtual surgery was performed to emulate the implemented surgical decisions, including removal of hip muscles and implantation of a custom prosthesis with total hip replacement.

**Results:** The participant’s post-surgery walking function was marked by a slower self-selected walking speed coupled with several compensatory mechanisms necessitated by lost or impaired hip muscle function, while the participant’s post-surgery neural control demonstrated a dramatic change in coordination strategy (as evidenced by modified time-invariant synergy vectors) with little change in recruitment timing (as evidenced by conserved time-varying synergy activations). Furthermore, the participant’s post-surgery muscle activations were fitted accurately using his pre-surgery synergy activations but fitted poorly using his pre-surgery synergy vectors.

**Discussion:** These results provide valuable information about which aspects of post-surgery walking function could potentially be improved through modifications to surgical decisions, custom prosthesis design, or rehabilitation protocol, as well as how computational simulations could be formulated to predict post-surgery walking function reliably given a patient’s pre-surgery walking data and the planned surgical decisions and custom prosthesis design.

## 1 Introduction

Pelvic sarcomas account for up to 20% of the approximately 2,500 primary bone tumor cases reported in the United States each year, where most patients are 25 years of age or younger (Morris, 2010; Mayerson et al., 2014). The complex anatomy of the pelvic region and the heterogeneity across patients in how tumors infiltrate the pelvis make surgical treatment for pelvic sarcomas challenging. Thanks to recent advances in medical imaging and multimodal oncological treatments (Puchner et al., 2017), internal hemipelvectomy surgery (Bickels and Malawer, 2001) has gained wider use for removing bone and soft tissue infiltrated by the tumor while sparing the limb. In pelvic sarcoma cases where the hip joint is infiltrated, the acetabulum and femoral head must also be removed. Initially, no reconstruction of the hip joint was the only surgical option. For this surgery, the surgeon wires the residual proximal femur into the residual pelvis, and during recovery, the limb is immobilized for approximately 12 months while scar tissue forms a flail hip joint. Following plateau in recovery, patients can walk well without a hip joint, but their gait pattern is abnormal, recovery time is long, and the risk of developing low back pain and scoliosis due to a limb-length discrepancy is high (Lackman et al., 2009; Wedemeyer and Kauther, 2011). More recently, custom prosthesis reconstruction with a total hip replacement has become a viable surgical option. For this surgery, the surgeon replaces the resected pelvic bone with a custom metal prosthesis and the resected hip joint with a total hip replacement, and during recovery, the limb is immobilized for only 3 months while bone grows into the metallic prosthetic components. Following plateau in recovery, patients can walk with a more normal gait pattern, have fewer functional limitations, and have no limb-length discrepancy, reducing the risk of developing low back pain and scoliosis (Lewis, 2014; Chao et al., 2015).

Unfortunately, few experimental studies have assessed how well custom prosthesis reconstruction with a total hip replacement is able to restore pre-surgery walking function and neural control. One study reported peak vertical ground reaction forces post-surgery to quantify the effects of an external hip stabilizing device on gait symmetry (Akiyama et al., 2016). A second study measured joint motion and metabolic energy expenditure to assess walking function following a two-year rehabilitation program (Wingrave and Jarvis, 2019). A third study collected comprehensive post-surgery gait data including vertical ground reaction forces, joint motions, joint moments, and electromyography (EMG) data to assess motor performance and walking (Valente et al., 2022). In all three studies, operated side biomechanical quantities were compared to non-operated side quantities following surgery, since no pre-surgery data were available from the same patients for comparison. Furthermore, while two studies reported extensive side-to-side comparisons of joint motion data, no study has presented ground reaction data for all three force components or joint moment data for all lower body joints. In addition, no study to date has quantified how a patient’s neural control strategy changes in response to the surgery. Since the surgery involves removal of multiple hip muscles, one might expect that a significant change in neural control would be required to allow a patient to walk following such extensive surgery. Even if comprehensive experimental walking data were available before and after surgery from the same patient to quantify changes in walking function, such data alone would not provide an objective means for improving surgical planning or custom pelvic prosthesis design.

Coupling comprehensive human movement data collection with personalized neuromusculoskeletal computer modeling provides an objective approach for quantifying changes in walking function as well as predicting how surgical decisions and custom prosthesis design will affect post-surgery walking function. Several previous studies have collected comprehensive human movement data sets from individuals who were implanted with an instrumented total knee replacement (Taylor et al., 2004; Fregly et al., 2012; Bergmann et al., 2014) or who suffered a stroke (Meyer et al., 2016; 2017; Sauder et al., 2019; Santos et al., 2021). These data sets include surface marker motion, ground reaction, and EMG data collected for walking and other functional tasks, and researchers have created associated personalized neuromusculoskeletal models using such data sets. Personalized models have been used to analyze joint contact loads in knee osteoarthritis, muscle force generation in cerebral palsy, and neural control capabilities in stroke (Fregly et al., 2012; Meyer et al., 2016; Saxby et al., 2016; Pitto et al., 2019; Kainz and Jonkers, 2023; Jing et al., 2023). They have also been used to develop predictive simulations of walking that investigate how an individual post-stroke would walk at his fastest comfortable speed (Meyer et al., 2016), how an individual post-stroke would walk while receiving functional electrical stimulation of different leg muscles (Sauder et al., 2019; Santos et al., 2021), and how a neurologically healthy individual would walk following different pelvic cancer surgeries with different levels of post-surgery rehabilitation (Vega et al., 2022). However, no study to date has validated a predictive simulation of post-surgery walking using post- and pre-surgery experimental walking data collected from the same patient, where the post-surgery data are used to validate the walking prediction and the pre-surgery data are used to create the personalized neuromusculoskeletal model of the patient.

While nearly all published three-dimensional predictive simulations of walking have used individual muscle controls (Anderson and Pandy, 2001; Fox et al., 2009; Koelewijn and van den Bogert, 2016; Lin et al., 2018; Song and Geyer, 2018; Falisse et al., 2019; Miller and Esposito, 2021; Santos et al., 2021; Cseke et al., 2022; Hu et al., 2022; Bianco et al., 2023), several studies have explored using muscle synergy controls as an alternative (Meyer et al., 2016; Pitto et al., 2019; Sauder et al., 2019; Vega et al., 2022). Muscle synergies (also called “motor modules” (Clark et al., 2010; Oliveira et al., 2014)) are a low-dimensional representation of a higher-dimensional control space (D’Avella et al., 2003; Tresch et al., 2006; Chvatal and Ting, 2013) and have traditionally been used to study how the healthy and impaired human central nervous system reduces control complexity for functional tasks such as walking. Muscle synergies are calculated by decomposing a large number (typically between 8 and 32) of processed experimental EMG signals (henceforth called “muscle excitations”) into a smaller number (typically between 3 and 7) of independent control signals (henceforth called “synergy excitations”) (Ivanenko et al., 2004; Cappellini et al., 2006; Clark et al., 2010; Torres-Oviedo and Ting, 2010), most commonly using a nonlinear optimization method called non-negative matrix factorization (Tresch et al., 2006; Rabbi et al., 2020). Each time-varying synergy excitation (also called an “activation signal” (Gizzi et al., 2011), “activation timing profile” (Clark et al., 2010), “activation profile” (Neptune et al., 2009), or “module pattern” (McGowan et al., 2010)) possesses a corresponding time-invariant synergy vector (also called a “muscle weighting” (Clark et al., 2010) or “motor module” (Gizzi et al., 2011)) containing weights that define how the synergy excitation contributes to each muscle excitation. The combination of a single synergy excitation and its associated synergy vector is termed a muscle synergy (Meyer et al., 2016; Banks et al., 2017; Sauder et al., 2019; Ao et al., 2020; Li et al., 2022; Vega et al., 2022). Because neural control is easier to study in a low-dimensional space, muscle synergies have been used to quantify differences in neural control between healthy and impaired conditions, including stroke (Clark et al., 2010; Gizzi et al., 2011; Kautz et al., 2011), cerebral palsy (Steele et al., 2015; Tang et al., 2015), Parkinson’s disease (Rodriguez et al., 2013), and knee osteoarthritis (Kubota et al., 2021; Taniguchi et al., 2024), as well as to quantify changes in neural control between pre- and post-treatment conditions for stroke (Routson et al., 2013) and cerebral palsy (Patikas et al., 2007; Shuman et al., 2019; Pitto et al., 2020). Furthermore, because neural control is also easier to model in a low-dimensional space, muscle synergies may also be useful as controls when developing predictive simulations of post-surgery walking function, especially if a patient’s synergy excitations or synergy vectors remain unchanged between pre- and post-treatment conditions.

This case study presents the most comprehensive quantitative assessment to date of walking function (ground reactions, joint motions, joint moments) and neural control (muscle synergies) changes produced by internal hemipelvectomy surgery with custom prosthesis reconstruction. The study combines collection of comprehensive pre- and post-surgery gait (video motion capture, ground reaction, EMG) and imaging (CT) data from a single participant with development of pre- and post-surgery personalized neuromusculoskeletal computer models of the same participant. The pelvic anatomy, joint functional axes, muscle-tendon properties, and muscle synergy controls in each personalized model were calibrated to the participant’s gait and imaging data obtained at the associated time point, with the post-surgery model accounting for the surgical decisions made by the orthopedic oncologist, including removal of hip muscles and implantation of a custom prosthesis with total hip replacement. The personalized models made it possible to analyze not only biomechanical changes, which provide valuable information about which aspects of post-surgery walking function remain abnormal and thus should be the targets for physical therapy, but also neural control changes, which provide valuable information about how muscle synergies could be used to develop predictive simulations of post-surgery walking function given pre-surgery walking data and the planned surgical decisions.

## 2 Methods

### 2.1 Experimental data collection

Experimental walking and CT scan data were collected pre- and post-surgery from a single participant with a pelvic sarcoma who received internal hemipelvectomy surgery with custom prosthesis reconstruction. The participant (sex: male, age: 46 years at the time of the surgery, height: 1.73 m and mass: 82.5 kg for both data collection sessions) gave written informed consent, and all data collection and subsequent computational analyses were approved by the institutional review boards of MD Anderson Cancer Center, the University of Texas Health Science Center Houston, and Rice University. The surgery involved resection of the tumor, hip joint, and surrounding muscles in the public and acetabular regions of the right hemipelvis, and the custom prosthesis included a total hip replacement. Pre-surgery data were collected the day before surgery, while post-surgery data were collected approximately 12 months after surgery once the participant had reached a plateau in recovery and could walk well without any assistive device.

Pre- and post-surgery walking and CT scan data were collected using identical protocols. Experimental walking data, including ground reaction, video motion capture, and EMG data, were collected while the participant walked for 2 minutes on a treadmill at his self-selected speed (1.0 m/s pre-surgery and 0.5 m/s post-surgery). In addition, a static standing trial was performed to facilitate a subsequent musculoskeletal model scaling operation (see below). Ground reaction data were collected using a split-belt instrumented treadmill (Bertec Corp., Columbus, OH, US) with belts tied. Video motion capture data were collected using an optical motion capture system (Qualisys AB, Gothenburg, Sweden). A total of 36 retroreflective markers were placed on the feet, legs, pelvis, torso, and arms consistent with a previous study (Meyer et al., 2016). Ground reaction and video motion capture data were low-pass filtered using a fourth-order zero-phase lag Butterworth filter with variable cut-off frequency dependent on the gait period (Hug, 2011). EMG data were collected from 15 lower extremity muscles per leg (Table 1) using both surface and fine wire electrodes (Cometa, Bareggio, Italy). EMG data processing was consistent with a previous study (Meyer et al., 2017), including high-pass filtering at 40 Hz, demeaning, rectifying, and low-pass filtering using a variable cut-off frequency dependent on the gait period (Hug, 2011). CT scan data of the participant’s entire pelvis and both proximal femurs were collected to facilitate the creation of pre- and post-surgery musculoskeletal models with personalized geometry. In addition, a geometric model of the participant’s custom pelvic prosthesis was made available by the orthopedic implant manufacturer (Onkos Surgical, Parsippany, NJ, US).

**TABLE 1:** List of lower extremity muscles in the musculoskeletal model, the source from which the muscle excitations were acquired and the acquisition method. The shaded muscles were surgically removed from the operated (right) leg during surgery.

Muscle names (Abbreviation)	Muscle excitation source	Muscle excitation acquisition method
Adductor brevis (ADB)	Adductor longus	Measured
Adductor longus (ADL)		
Adductor magnus distal (ADM1)		
Adductor magnus ischial (ADM2)		
Adductor magnus middle (ADM3)		
Adductor magnus proximal (ADM4)		
Gracilis (GRAC)		
Biceps femoris long head (BFLH)	Biceps femoris long head	
Biceps femoris short head (BFSH)		
Gastrocnemius lateral (GL)	Gastrocnemius lateral	
Gastrocnemius medial (GM)	Gastrocnemius medial	
Gluteus maximus superior (GMA1)	Gluteus maximus	
Gluteus maximus middle (GMA2)		
Gluteus maximus inferior (GMA3)		
Gluteus medius anterior (GME1)	Gluteus medius	
Gluteus medius middle (GME2)		
Gluteus medius posterior (GME3)		
Gluteus minimus anterior (GMI1)		
Gluteus minimus middle (GMI2)		
Gluteus minimus posterior (GMI3)		
Iliacus (IL)	Iliopsoas^a^	
Psoas major superior (PS1)		
Psoas major middle (PS2)		
Psoas major inferior (PS3)		
Peroneus brevis (PB)	Peroneus	
Peroneus longus (PL)		
Rectus femoris (RF)	Rectus femoris	
Pectineus (PECT)		
Semimembranosus (SM)	Semimembranosus	
Semitendinosus (ST)		
Vastus medialis (VM)	Vastus medius	
Vastus intermedius (VI)		
Vastus lateralis (VL)	Vastus lateralis	
Soleus (SOL)	Soleus	
Tibialis anterior (TA)	Tibialis anterior	
Tibialis posterior (TP)	Tibialis posterior	
Extensor digitorum longus (EDL)	Extensor digitorum longus	Synergy extrapolation
Extensor hallucis longus (EHL)		
Flexor digitorum longus (FDL)	Flexor digitorum longus	
Flexor hallucis longus (FHL)		
Gemellus (GEM)	Piriformis	
Piriformis (PIRI)		
Quadratus femoris (QF)		
Sartorius (SART)	Sartorius	
Tensor fasciae latae (TFL)	Tensor fasciae latae	

^a^ EMG measurement of the right iliopsoas was unavailable pre- and post-surgery.

### 2.2 Neuromusculoskeletal model analyses

Personalized neuromusculoskeletal computer models of the participant were constructed to represent pre- and post-surgery conditions, where all geometric musculoskeletal modeling was performed in OpenSim (Delp et al., 2007; Seth et al., 2018). The model personalization process started with a generic OpenSim model constructed by combining previously published lower extremity models (Arnold et al., 2010; Rajagopal et al., 2016; Lai et al., 2017) and lumbar-spine models (Christophy et al., 2012; Bruno et al., 2015). This process was performed to produce a new generic model that possessed all of the muscles required for the present study. The resulting generic model possessed 45 muscles in each leg to actuate the following rotational joints with their associated number of degrees of freedom: hip (3), knee (1), ankle (1), subtalar (1), and toes (1) on each leg.

Starting from this generic model, we constructed a pre-surgery model of the participant using his pre-surgery walking and CT scan data as described in a previous study (Li et al., 2022). In brief, the body segments in the generic model were scaled to match the participant using static trial motion capture data and the OpenSim Model Scaling Tool. The one exception was the pelvis, whose dimensions were scaled separately in all three directions to match the participant’s pelvis geometry as determined from pre-surgery CT scan data. The scaled generic pelvis geometry was then replaced with personalized pelvis geometry constructed from pre-surgery CT scan data. Pelvis muscle attachment locations were adjusted to be on bony anatomy by following a codified workflow (Modenese et al., 2018) using nmsBuilder (Valente et al., 2017).

**FIGURE 1:**
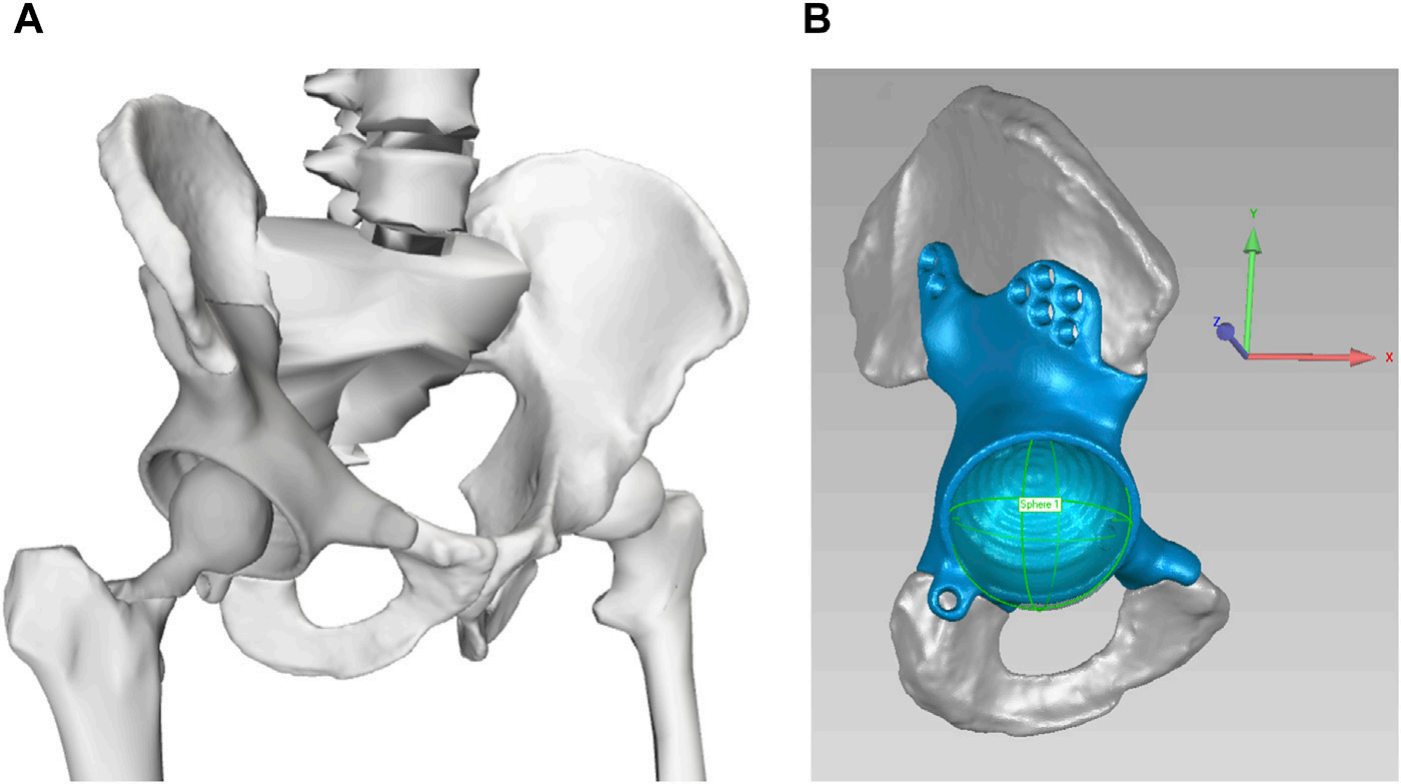
**(A)** Geometric model of the remaining pelvic bone, custom prosthesis, and total hip replacement. **(B)** Updated hip joint center on the operated side, as determined from the center of the sphere used to fit the inner surface of the acetabular component. The coordinate system is consistent with OpenSim coordinate system: +x points in anterior direction, +y points in the superior direction, and +z points in the lateral (right) direction.

Starting from the pre-surgery model, we then constructed a post-surgery model of the participant by modifying his pre-surgery model to account for implantation of a custom pelvic prosthesis and the surgical decisions made by the orthopedic oncologist. To account for implantation of a custom pelvic prosthesis, we developed a bone-prosthesis geometric model of the participant’s pelvis and operated femur. For the pelvis with custom prosthesis, we first used a two-step process of global registration and fine alignment in Geomagic Wrap (3D Systems, Morrisville, NC, US) to align a post-surgery geometric model of the pelvis plus custom prosthesis with the pre-surgery geometric model of the pelvis. The pre-surgery pelvis geometric model was subsequently replaced by the post-surgery pelvis-prosthesis geometric model (Figure 1A). The hip joint center on the operated side was updated with the center of a sphere used to fit the inner surface of the acetabular component (Figure 1B). For the post-surgery femur with femoral component, we obtained the bone and implant geometry from post-surgery CT scan data. The femoral geometry in the pre-surgery model on the operated side was then re-scaled only in the superior-inferior direction to account for the slight change in femur length due to implantation of the femoral component. After the post-surgery femur with femoral component geometry was aligned to the re-scaled pre-surgery femoral geometry using Geomagic Wrap, the re-scaled pre-surgery femur geometry was replaced with the post-surgery femur with femoral component geometry. To account for the surgical decisions made by the orthopedic oncologist, we set to zero the peak isometric strength of 15 muscle heads surgically removed from the operated leg (see Supplementary Table S1). For muscles that were surgically detached and reattached, the pre-surgery peak isometric strength and geometry were retained in the post-surgery model.

A sequence of three standard OpenSim operations were performed using the participant’s pre- and post-surgery musculoskeletal models to quantify changes in gait biomechanics and to generate the input data needed for EMG-driven model calibration (Lloyd and Besier, 2003; Buchanan et al., 2004; Sartori et al., 2014). The OpenSim Inverse Kinematics Tool was used to compute joint kinematics using the participant’s motion capture marker data. The OpenSim Inverse Dynamics Tool was used to compute joint moments using the participant’s calculated joint kinematics and experimental ground reaction data. The OpenSim Muscle Analysis Tool was used to compute muscle-tendon lengths and muscle moment arms using the participant’s joint kinematics. Ten pre- and post-surgery gait cycles were selected that possessed the smallest root-mean-square error (RMSE) values for joint angle and joint moment curves with respect to corresponding mean curves. This selection process eliminated outlier gait cycles while maintaining comparable levels of inter-cycle variability between the two test sessions. Data from these gait cycles were used for all subsequent analyses.

Using data from these ten pre- and post-surgery gait cycles, we solved a multi-objective optimization problem to calibrate an EMG-driven neuromusculoskeletal model for the hip, knee, ankle, and subtalar joints of each leg before and after surgery (Meyer et al., 2017). The design variables adjusted by the multi-objective optimization were the following: 1. Activation dynamics model parameters: EMG scale factors, electromechanical delays, activation time constants, and activation non-linear shape factors (He et al., 1991; Lloyd and Besier, 2003), 2. Hill-type muscle-tendon model parameters: optimal fiber length and tendon slack length (Zajac, 1989), and 3. Synergy vector weights for constructing residual and predicted muscle excitations from experimental synergy excitations. Residual excitations are small changes applied to experimental muscle excitations that enable more accurate estimation of predicted muscle excitations (Ao et al., 2022). Peak isometric strength parameter values for all lower extremity muscle-tendon models were calculated using published regression relationships derived from MRI data (Handsfield et al., 2014). The primary cost function term minimized the sum of squares of differences between experimental joint moments calculated via inverse dynamics and model joint moments calculated using the patient’s personalized neuromusculoskeletal model. Secondary cost function terms minimized the sum of squares of residual and predicted muscle excitations.

Within the EMG-driven model calibration process, synergy extrapolation (Bianco et al., 2018; Ao et al., 2020; Ao et al., 2022; Ao et al., 2023) was utilized to estimate missing EMG signals. Synergy extrapolation uses synergy excitations extracted from muscles with experimental EMG data to predict muscle excitations for muscles without experimental EMG data (Table 1) (a detailed explanation of how synergy extrapolation works can be found in Ao et al. (2022)). Synergy extrapolation was chosen over static optimization since it has been shown to estimate missing EMG signals more reliably than does static optimization (Ao and Fregly, 2024).

The EMG-driven model calibration process was first performed for each leg separately, and each calibrated model yielded activations dynamics and muscle-tendon model parameter values along with estimates of muscle excitations and activations, henceforth referred to collectively as “muscle controls.” Muscle activations were calculated by applying electromechanical delays and activation dynamics to the muscle excitations after they were normalized as part of the EMG-driven model calibration process. The entire model personalization process required several months of effort, including the time needed to learn new software tools and computational methodologies.

### 2.3 Lower extremity muscle synergy analyses

The pre- and post-surgery muscle controls for each leg were decomposed into a lower dimensional space using muscle synergy analysis (MSA). MSA was performed via non-negative matrix factorization (NMF) (Tresch et al., 2006; Rabbi et al., 2020) using the MATLAB ‘nnmf’ function (MathWorks, Natick, MA) on both muscle excitations and muscle activations for each pre- and post-surgery gait cycle using only muscles with experimental EMG data (Table 1). Although MSA is typically performed on only muscle excitations, this study performed MSA on muscle activations as well since they possess different timings (due to electromechanical delays) and shapes (due to activation dynamics) compared to muscle excitations, potentially altering MSA results. Each muscle synergy consisted of a time-varying synergy control along with a corresponding time-invariant synergy vector containing muscle-specific weights. Synergy controls provided information about recruitment timing (i.e., when groups of muscles were co-activated) while synergy vectors provided information about coordination strategy (i.e., how groups of muscles were co-activated). Synergy controls extracted from muscle excitations were referred to as synergy excitations, while those extracted from muscle activations were referred as synergy activations . MSA was performed assuming 4, 5, 6 and 7 synergies for each leg as this range covers the typical number of synergies required to adequately represent muscle activity during gait (Ivanenko et al., 2004; Meyer et al., 2016). The variability accounted for (VAF) metric was used to quantify how well the calculated muscle synergies could represent the original muscle controls. Since muscle synergies were calculated for each gait cycle separately and the NMF algorithm does not output muscle synergies in any particular order, muscle synergies were sorted based on cosine similarity between synergy vectors across gait cycles (Banks et al., 2017).

To account for how a difference between pre- and post-surgery walking speed affected the magnitude of the synergy controls (Clark et al., 2010), we introduced a new magnitude quantity into the muscle synergy decomposition equation (Eq. 1):

$$C\overset{NMF}{\to }\sum_{i=1}^{s}C_{syn_{i}}∙w_{sy{n\;norm}_{i}}=\sum_{i=1}^{s}C_{syn\;norm_{i}}∙w_{syn\;norm_{i}}∙M_{syn_{i}}$$ (1)

where C are muscle controls, NMF indicates application of non-negative matrix factorization, $C_{syn}$ are the time-varying synergy controls, and $w_{syn\;norm}$ are the time-invariant normalized synergy vectors. The dimensions of the muscle controls C , synergy controls $C_{syn}$ , and synergy vectors, $w_{syn\;norm}$ are *n× s*, *n× m*, and *s× m*, respectively, where *n* is the number of data points used in a normalized gait cycle, *m* is the number of muscles, and *s* is the number of muscle synergies. Synergy vectors are normalized automatically to a magnitude of one by the Matlab ‘nnmf’ non-negative matrix factorization algorithm, but synergy controls are not normalized. Consequently, we normalized each synergy control to a maximum value of one and added a new parameter $M_{syn}$ to represent the magnitude of the synergy. With this modification, synergy controls provide information about only recruitment timing, synergy vectors information about only coordination strategy, and synergy magnitudes information about only synergy magnitude, making it easier to quantify synergy changes produced by changes in walking speed.

Since all muscle synergies were calculated using muscle excitations and activations produced by a calibrated EMG-driven lower extremity model, the resulting muscle synergies where functional rather than merely descriptive. Standard MSA is applied to muscle excitations normalized by one of several common methods (e.g., maximum value from a maximum voluntary contraction trial, maximum value over all trials, unit variance). However, the way muscle excitations are normalized affects MSA results (Banks et al., 2017) and the net joint moments calculated from the normalized muscle excitations. Because standard MSA only involves accurately reconstructing experimental muscle excitations, it provides only descriptive information about the participant’s neural control strategy. The calculated muscle synergies will not produce the correct net joint moments when input into a personalized neuromusculoskeletal model of the participant. In contrast, MSA as performed in the present study involves accurately reconstructing experimental muscle excitations as well as accurately reproducing inverse dynamic joint moments, providing functional information about how the participant’s muscle synergies generate the experimentally measured net joint moments.

### 2.4 Post-surgery muscle control reconstruction

We explored three options for how post-surgery muscle excitations and activations could be reconstructed using corresponding pre-surgery muscle synergy information. For the first option, termed the Fixed Synergy Vector method, the post-surgery synergy vectors were assumed to be identical to the pre-surgery synergy vectors, implying the coordination strategy was conserved, and the corresponding post-surgery synergy controls needed to reconstruct the post-surgery muscle controls were calculated. For the second option, termed the Fixed Synergy Control method, the post-surgery synergy controls were assumed to be identical to the pre-surgery synergy controls, implying the recruitment timing was conserved, and the corresponding post-surgery synergy vectors required to reconstruct the post-surgery muscle controls were calculated. For the third option, termed the Shifted Synergy Control method, the post-surgery synergy controls were assumed to be identical to the pre-surgery synergy controls except with small time shifts, implying the recruitment timing was conserved apart from small time shifts, and the corresponding post-surgery synergy vectors required to reconstruct the post-surgery muscle controls were calculated.

We implemented all three options for reconstructing post-surgery muscle controls using the MSA results obtained for the pre-surgery muscle controls. For the Fixed Synergy Vector method, the mean synergy vectors across all pre-surgery gait cycles were defined as the fixed synergy vectors, and the synergy controls required to reconstruct the post-surgery muscle controls for each gait cycle were found (Eq. 2). For the Fixed Synergy Control method, the mean synergy controls across all pre-surgery gait cycles were defined as the fixed synergy controls, and the synergy vectors required to reconstruct the post-surgery muscle controls for each gait cycle were found (Eq. 3). For the Shifted Synergy Control method, each pre-surgery synergy control was shifted in time to maximize its cosine similarity with the corresponding post-surgery synergy control (Supplementary Figure S5). The shifted pre-surgery synergy controls were then used as the fixed synergy controls in Eq. 3, and the synergy vectors required to reconstruct the post-surgery muscle controls for each gait cycle were found. Synergy controls and vectors were considered to be non-negative and therefore nonnegative linear least-squares optimization problems were solved using the MATLAB ‘lsqnonneg’ function. To calculate synergy controls for the Fixed Synergy Vector method, we used the following problem formulation:

$$\begin{array}{c} \min_{C_{syn_{fit}}}{\left\|C_{syn_{fit}}∙w_{syn_{pre}}-C_{{mus}_{post}}\right\|}^{2}\\ \text{subject to}\\ C_{syn_{fit}}\geq 0 \end{array}$$ (2)

where $C_{syn_{fit}}$ are the synergy control design variables for each gait cycle that produce the best fit of $C_{{mus}_{post}}$ , the post-surgery muscle controls for each gait cycle, using $w_{syn_{pre}}$ , the mean pre-surgery synergy vectors. Similarly, to calculate synergy vectors for the Fixed or Shifted Synergy Control method, we solved:

$$\begin{array}{c} \min_{w_{syn_{fit}}}{\left\|C_{syn_{pre/shifted}}∙w_{syn_{fit}}-C_{{mus}_{post}}\right\|}^{2}\\ \text{subject to}\\ w_{syn_{fit}}\geq 0 \end{array}$$ (3)

where $w_{syn_{fit}}$ are the synergy vector design variables for each gait cycle that produce the best fit of $C_{{mus}_{post}}$ , the post-surgery muscle controls for each gait cycle, using $C_{syn_{pre/shifted}}$ , the mean pre-surgery synergy controls without or with time shifting.

To evaluate how well the post-surgery muscle controls were reconstructed, we calculated the variability accounted for (VAF) between reconstructed and experimental muscle controls for each post-surgery gait cycle. The evaluation was performed for each combination of the following three methodological choices: 1. selected reconstruction method (Fixed Synergy Vector, Fixed Synergy Control, or Shifted Synergy Control method), 2. type of muscle controls to be reconstructed (excitations or activations), and 3. number of synergies used per leg (4, 5, 6, or 7).

### 2.5 Statistical analyses

Statistical analyses were used to compare time-varying pre- and post-surgery biomechanical and neural control quantities and time-invariant VAF values for reconstructing post-surgery muscle controls from pre-surgery muscle synergies produced by different combinations of methodological choices (selected reconstruction methods, types of muscle controls reconstructed, and number of synergies used for reconstruction). For all statistical tests, the significance level was set at *p* < 0.05 prior to any correction for multiple comparisons, where the null hypothesis was that the quantities being compared were not statistically different.

To compare time-varying quantities (ground reaction forces, joint angles, joint moments, and synergy controls) before and after surgery, we used statistical parametric mapping (SPM) (Penny et al., 2011) employing random field theory to correct for multiple comparisons across time (Pataky, 2008). SPM presents statistical results in the same temporal space as the original data, making interpretation of results straightforward (Pataky, 2012). SPM used two-tailed two sample t-tests (*p* < 0.05) to compare pre- and post-surgery time-varying quantities across the gait cycle. SPM analyses were implemented using the ‘ttest2’ function from the opensource SPM code ‘spm1d’ in MATLAB (Pataky, 2012).

To compare time-invariant VAF values produced by different methodological choices for reconstructing post-surgery muscle controls from pre-surgery muscle synergies, we used paired sample *t*-tests implemented using the MATLAB ‘ttest’ function. Each sample consisted of ten VAF values, one for each post-surgery gait cycle, calculated using a specified methodological choice (e.g., Fixed Synergy Vector method applied to muscle excitations using 4 synergies). The statistical analyses were performed for the following three cases. Case 1: the two samples were obtained by using different reconstruction methods but the same type of reconstructed muscle controls and the same number of synergies. Case 2: the two samples were obtained by using different types of reconstructed muscle controls but the same reconstruction method and the same number of synergies. Case 3: the two samples were obtained by using different numbers of synergies but the same reconstruction method and the same type of reconstructed muscle controls. Since each case involved multiple comparisons (i.e., between different reconstruction methods, types of reconstructed muscle controls, and numbers of synergies), Bonferroni correction was used to adjust the level of statistical significance as appropriate for each case (see Supplementary Table S4 for adjusted *p*-values).

## 3 Results

### 3.1 Walking function changes

**FIGURE 2:**
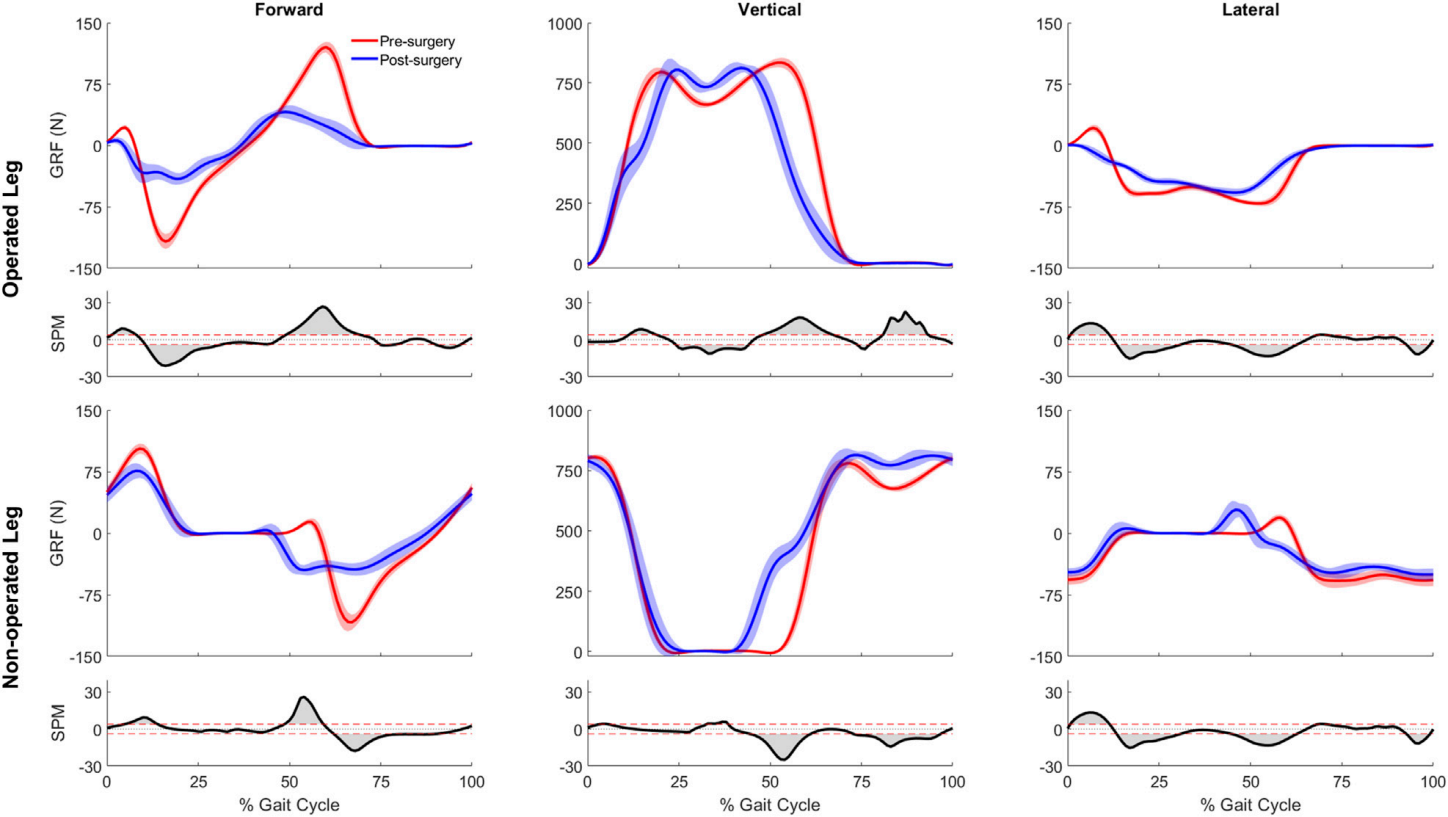
Pre- and post-surgery ground reaction forces (mean ±1 standard deviation across 10 gait cycles), along with SPM test results (grey shaded area indicates significant difference between pre- and post-surgery data).

Significant differences were observed between the pre- and post-surgery ground reaction, joint motion, and joint moment data obtained from the participant’s experimental walking data and personalized neuromusculoskeletal models. For ground reaction forces or GRF (Figure 2), the vertical GRF during the loading phase and mid-stance were different for the operated and non-operated legs between post and pre-surgery conditions, as was the vertical GRF during the unloading phase for the operated leg (*p* < 0.001). The decrease in vertical GRF during operated leg unloading and the increase in vertical GRF during non-operated leg loading each started earlier in the gait cycle during post-surgery walking, suggesting that the non-operated leg was compensating for the operated leg. Propulsive force shortly before toe-off and braking force following heel strike were also significantly lower post-surgery than pre-surgery for both legs (*p* < 0.001), possibly related to the decrease in self-selected walking speed post-surgery.

**FIGURE 3:**
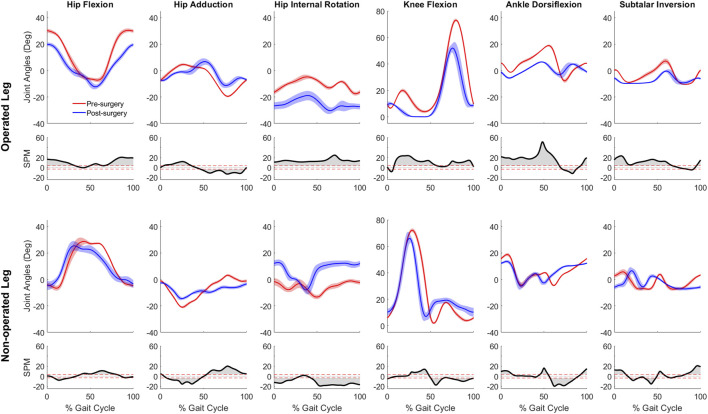
Pre- and post-surgery joint motions (mean ±1 standard deviation across 10 gait cycles) for all lower extremity joints, along with SPM test results (grey shaded area indicates significant difference between pre- and post-surgery data).

**FIGURE 4:**
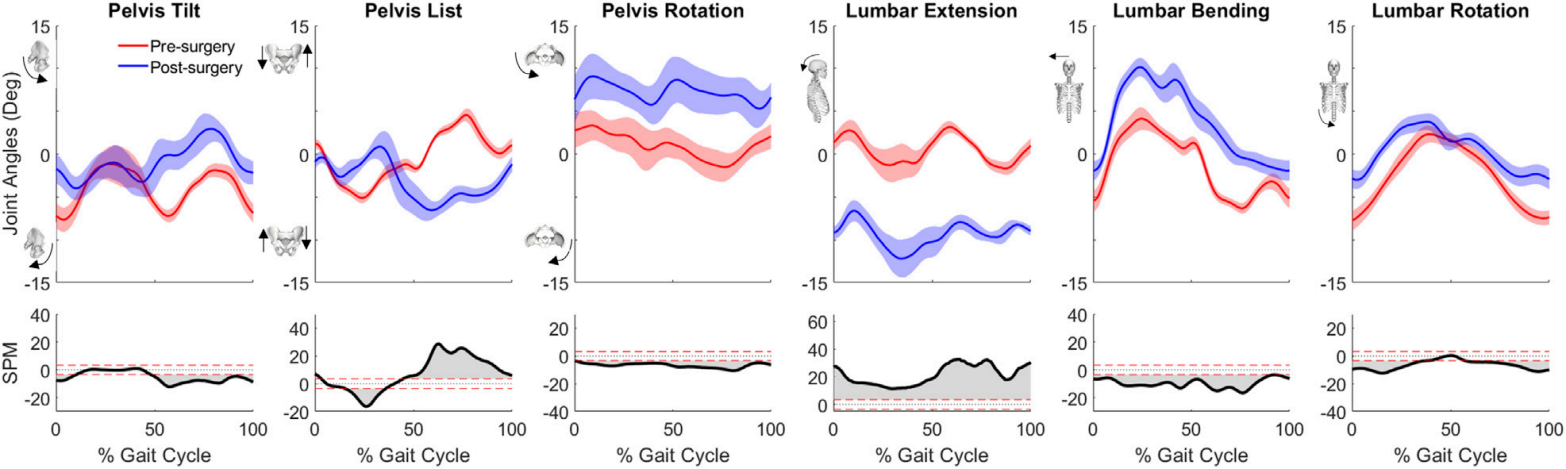
Pre- and post-surgery joint motions (mean ±1 standard deviation across 10 gait cycles) for pelvis and lumbosacral orientations, along with SPM test results (grey shaded area indicates significant difference between pre- and post-surgery data).

For joint motion data, all joint angle trajectories for both the operated and non-operated leg showed significant changes between pre- and post-surgery conditions (*p* < 0.001). For the operated leg following surgery (Figure 3), the hip was more extended and externally rotated over most of the gait cycle, the knee was less flexed over most of the gait cycle, and the ankle was less dorsiflexed and the subtalar joint less inverted during stance phase. In contrast, for the non-operated leg following surgery (Figure 3), the hip was more extended only during the first half of stance phase and more internally rotated over most of the gait cycle, the knee was more flexed during stance phase but less flexed during swing phase, the ankle was more dorsiflexed during the first half of stance phase, and subtalar joint exhibited reduced inversion in the middle of stance phase. For the pelvis and trunk (Figure 4), joint rotations generally became more asymmetric following surgery, with pelvis tilt losing its stereotypical double humped pattern, pelvis list showing a drop toward the non-operated side, pelvis rotation exhibiting an offset that moved the operated hip forward, lumbar extension demonstrating an increase in forward tilt, and lumbar bending increasing toward the operated side.

**FIGURE 5:**
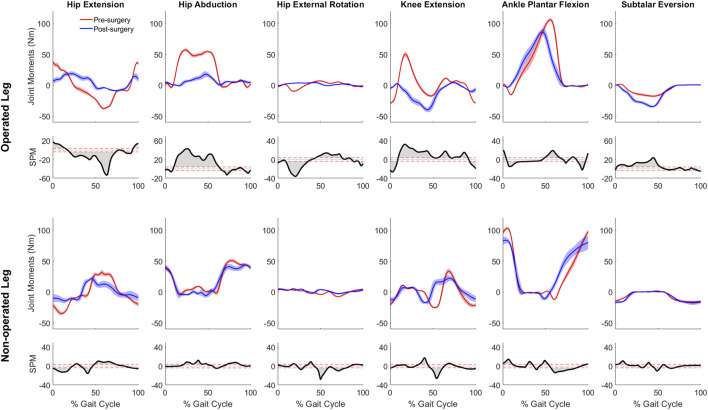
Pre- and post-surgery joint moments (mean ± standard deviation across 10 gait cycles) for all lower extremity joints, along with SPM test results (grey shaded area indicates significant difference between pre- and post-surgery data).

For joint moment data (Figure 5), more pronounced changes (*p* < 0.001) over larger portions of the gait cycle) occurred in the operated leg than in the non-operated leg following surgery. For the operated leg during stance phase, the hip flexion and abduction moments decreased, the knee extension moment turned into a flexion moment, and the subtalar inversion moment increased. Changes during swing phase were minimal. In contrast, for the non-operated leg, joint moment changes were minimal following surgery, with scattered statistically significant differences occurring at various points throughout the gait cycle.

### 3.2 Muscle-tendon model parameter changes

**FIGURE 6:**
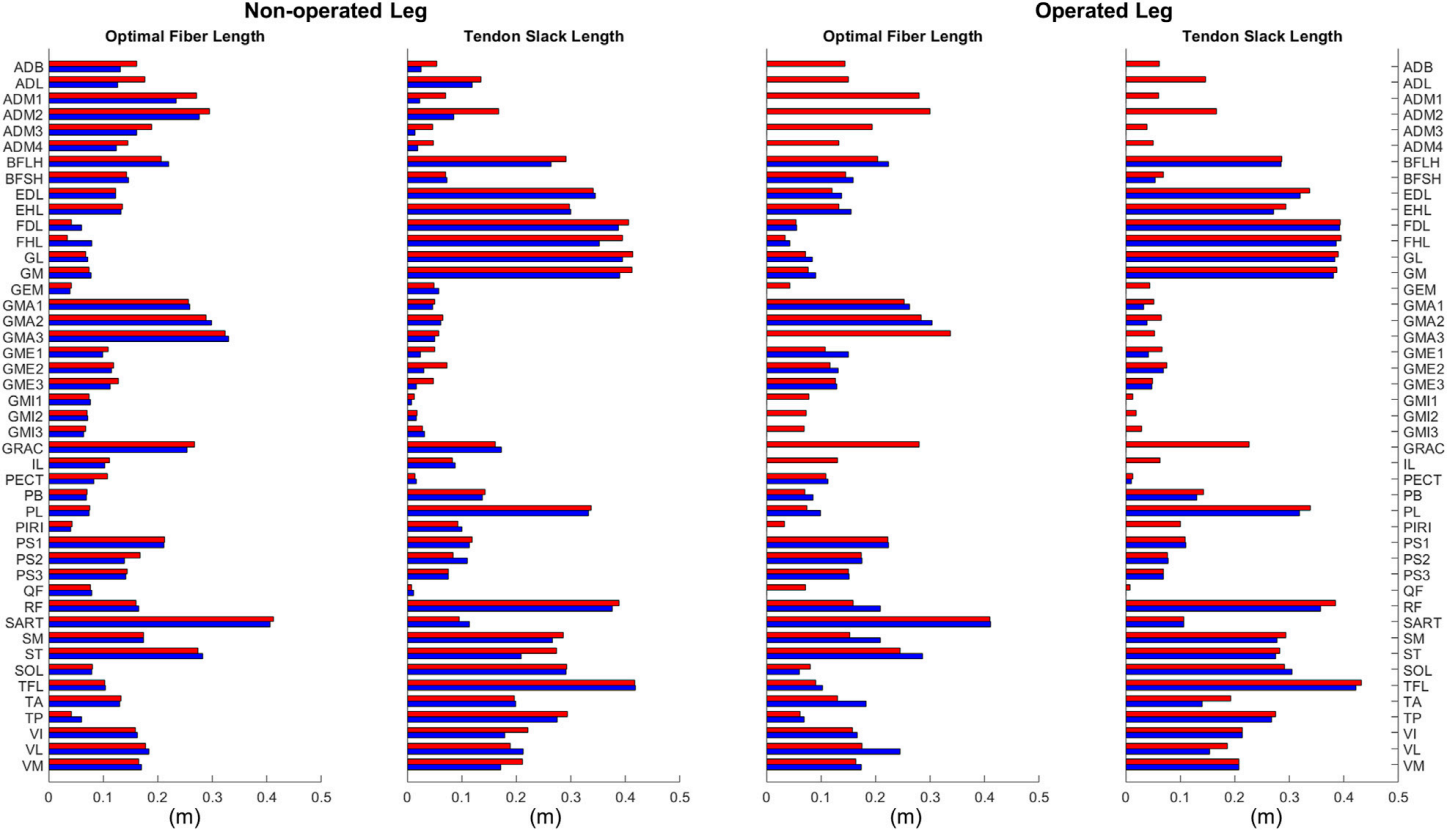
Optimal muscle fiber length and tendon slack length values for lower extremity muscles in the personalized pre-surgery (red) and post-surgery (blue) neuromusculoskeletal models.

Optimal muscle fiber length and tendon slack length values found by EMG-driven neuromusculoskeletal model calibration showed relatively small differences on average between pre- and post-surgery conditions (Figure 6). For optimal fiber length, the changes ranged from −0.019 to 0.070 m (mean 0.018 m and median 0.013 m) for the operated leg, and from −0.050 to 0.044 m (mean −0.003 m and median −0.0005 m) for the non-operated leg. In terms of percentages with respect to pre-surgery values, these changes ranged from −24% to 40% (mean 14% and median 13%) for the operated leg, and from −28% to 132% (mean 2% and median −1%) for the non-operated leg. For tendon slack length, the changes ranged from −0.051 to 0.013 m (mean −0.011 m and median −0.008 m) for the operated leg, and from −0.082 to 0.027 m (mean −0.013 m and median −0.005 m) for the non-operated leg. In terms of percentages with respect to pre-surgery values, the changes ranged from −40% to 5% (mean −9% and median −3%) for the operated leg, and from −70% to 43% (mean −11% and median −5%) for the non-operated leg.

### 3.3 Neural control changes

**FIGURE 7:**
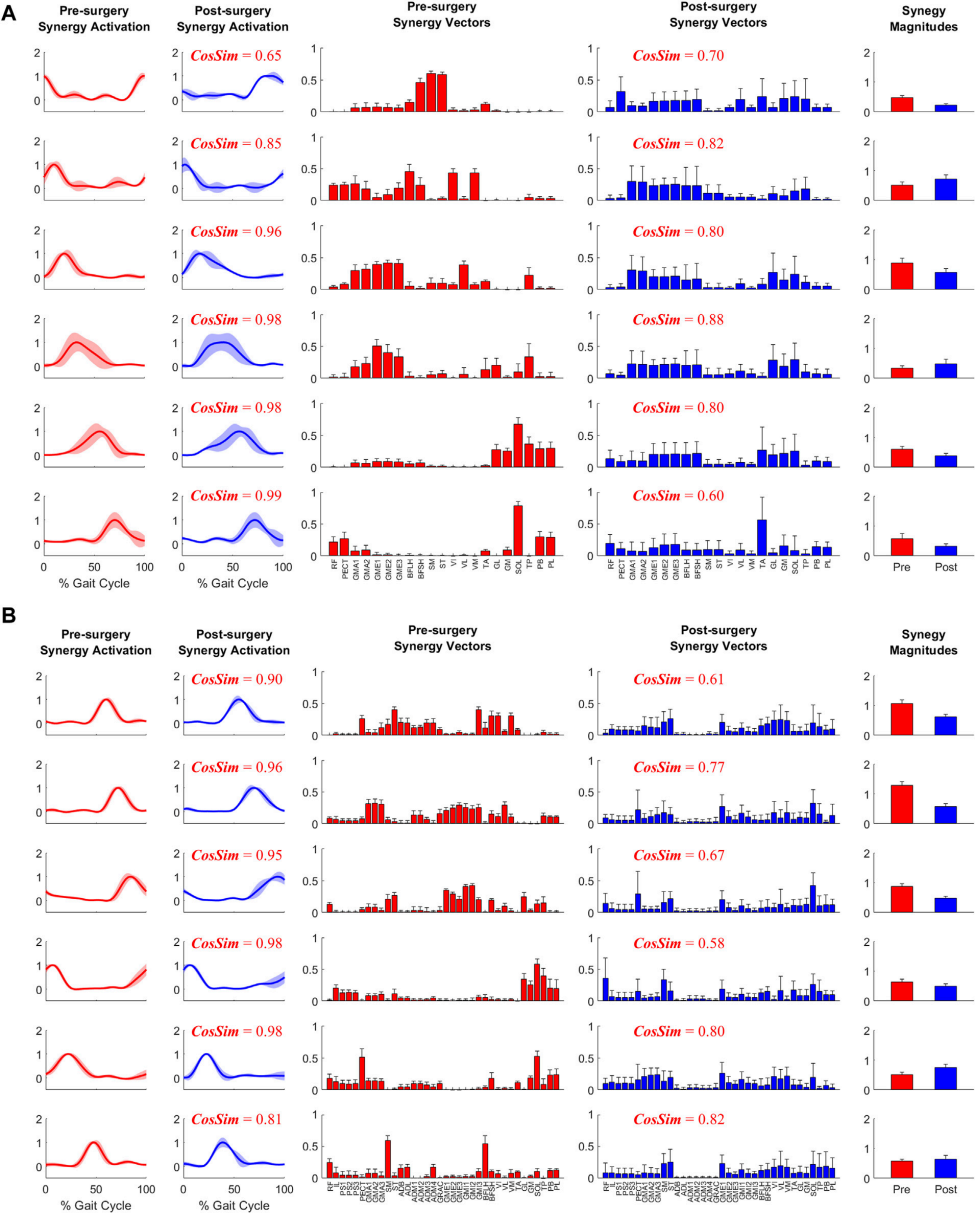
Example plot of pre- and post-surgery muscle synergies (mean ±1 standard deviation across 10 gait cycles) for **(A)** Operated Leg and **(B)** Non-operated Leg. Cosine similarity was calculated using the mean values for each pair of synergy activations or synergy vectors.

When post-surgery muscle synergies were compared to pre-surgery muscle synergies, stronger similarities were found between synergy excitations or activations than between synergy vectors (Table 2). The cosine similarity values between the pre- and post-surgery synergy controls were generally higher than those between the synergy vectors for both the operated and non-operated leg. When the pre-surgery synergy controls were shifted to maximize cosine similarity (Supplementary Figure S5), the cosine similarity values between the optimally shifted pre-surgery and post-surgery synergy controls were close to one (Table 2), indicating extremely strong similarity. The shifts required to achieve maximum cosine similarity (Supplementary Table S3) appeared to decrease with an increasing number of synergies. Figure 7 illustrates the comparison between paired pre- and post-surgery muscle synergy quantities and how cosine similarity values were calculated between paired synergy activations and synergy vectors.

**TABLE 2:** Mean ± standard deviation of cosine similarity between pre- and post-surgery muscle synergy quantities across synergies. For each pair of pre- and post-surgery muscle synergies identified after sorting and pairing, we calculated cosine similarity between (1) mean muscle synergy control curves without shifting, (2) mean muscle synergy control curves with shifting, and (3) mean synergy vectors. Mean synergy controls and synergy vectors were calculated from all ten pre- or post-surgery gait cycles analyzed. For (2), the mean pre-surgery synergy controls were shifted to maximize similarity to the mean post-surgery synergy controls. MSA was performed using both muscle excitations and muscle activations to determine potential differences caused by the type of muscle control analyzed.

Number of synergies	Muscle control	Cosine similarity					
		Non-operated leg			Operated leg		
		(1) Synergy controls	(2) Shifted controls	(3) Synergy vectors	(1) Synergy controls	(2) Shifted controls	(3) Synergy vectors
4	Excitation	0.77 ± 0.18	0.95 ± 0.03	0.71 ± 0.11	0.90 ± 0.08	0.95 ± 0.03	0.75 ± 0.15
	Activation	0.84 ± 0.11	0.94 ± 0.02	0.78 ± 0.05	0.74 ± 0.25	0.98 ± 0.01	0.68 ± 0.20
5	Excitation	0.90 ± 0.05	0.95 ± 0.05	0.83 ± 0.09	0.86 ± 0.12	0.96 ± 0.05	0.73 ± 0.10
	Activation	0.89 ± 0.05	0.95 ± 0.02	0.75 ± 0.09	0.84 ± 0.19	0.98 ± 0.02	0.72 ± 0.14
6	Excitation	0.94 ± 0.03	0.97 ± 0.02	0.79 ± 0.05	0.87 ± 0.05	0.95 ± 0.05	0.76 ± 0.08
	Activation	0.93 ± 0.06	0.98 ± 0.01	0.71 ± 0.10	0.90 ± 0.13	0.97 ± 0.02	0.77 ± 0.10
7	Excitation	0.76 ± 0.18	0.96 ± 0.02	0.78 ± 0.04	0.91 ± 0.12	0.97 ± 0.02	0.77 ± 0.09
	Activation	0.94 ± 0.07	0.98 ± 0.01	0.81 ± 0.08	0.95 ± 0.03	0.97 ± 0.03	0.82 ± 0.05

### 3.4 Post-surgery muscle control predictions

**FIGURE 8:**
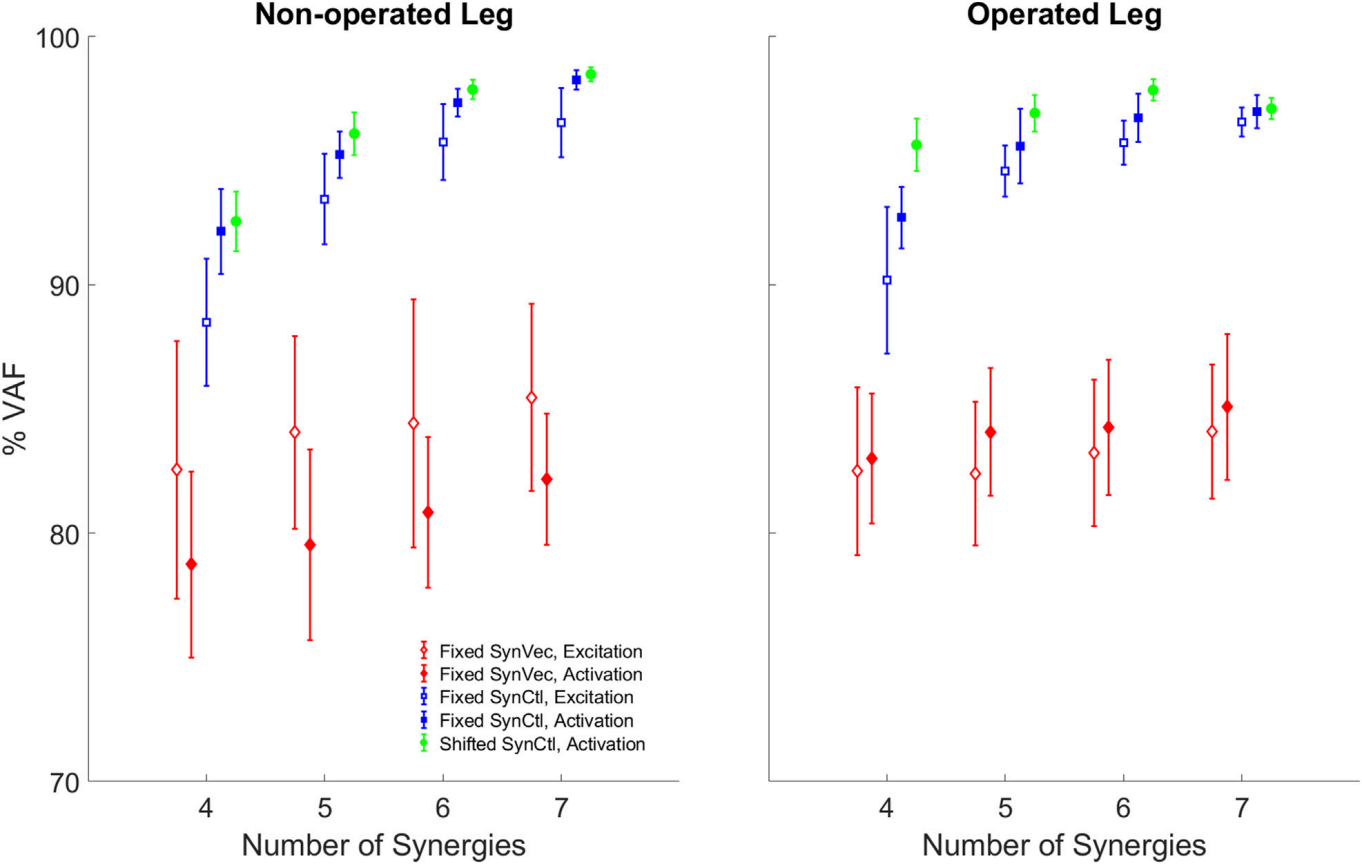
Variability accounted for (VAF) by reconstructed post-surgery muscle controls using each method: FixedSynVec (Fixed Synergy Vector, red), FixedSynCtl (Fixed Synergy Control, blue), and ShiftedSynCtl (Shifted Synergy Control, green). Each marker with error bars indicates mean ±1 standard deviation for VAF values from 10 gait cycles. Open markers indicate reconstruction of muscle excitations while filled markers indicate reconstruction of muscle activations.

When pre-surgery muscle synergy quantities were used to fit post-surgery muscle controls, better fits were obtained using pre-surgery synergy controls rather than pre-surgery synergy vectors (Figure 8). Both the Fixed Synergy Control and Shifted Synergy Control method achieved higher VAF than did the Fixed Synergy Vector method for fitting post-surgery muscle excitations and activations with any number of synergies between 4 and 7 (*p* < 0.003125). The Shifted Synergy Control method applied to muscle activations achieved higher VAF than did the corresponding Fixed Synergy Control method using 5 or 6 synergies for the non-operated leg (*p* < 0.00625) and 4, 5, or 6 synergies for the operated leg (*p* < 0.00625). The Fixed Synergy Control method performed significantly better when applied to muscle activations rather than muscle excitations (*p* < 0.00625) except for the operated leg with 7 synergies (*p* = 0.0591). The performance of both the Fixed Synergy Control and Shifted Synergy Controls methods applied to muscle activations generally improved with increasing number of synergies (*p* < 0.0125), except when the number of synergies increased from 6 to 7 for the operated leg (*p* = 0.1065).

## 4 Discussion

This study analyzed changes in walking function and neural control for a single pelvic sarcoma patient after internal hemipelvectomy surgery with custom prosthesis reconstruction. Personalized neuromusculoskeletal computational models representing the participant before and after surgery were developed to quantify changes in biomechanical quantities, neuromusculoskeletal model parameter values, and neural control quantities due to the surgery. Muscle synergy analyses were performed on the experimental muscle excitations and associated muscle activations for lower extremity muscles with experimental EMG data to quantify changes in neural control. Three methods for predicting post-surgery muscle controls using pre-surgery muscle synergy information were evaluated. Our findings suggest that for the participant analyzed in this study, post-surgery walking function differs substantially from pre-surgery walking function despite a post-surgery gait pattern that does not appear visually to be highly abnormal. These quantified differences could provide targets for personalized rehabilitation efforts for this participant. Furthermore, our findings suggest that the Fixed and Shifted Synergy Control methods reconstructed post-surgery muscle controls better than did the Fixed Synergy Vector method. The total VAF for reconstructed post-surgery muscle activations reached 95% using only five synergies for the Shifted Synergy Control method and six synergies for the Fixed Synergy Control method. Consequently, future studies that seek to use personalized computational neuromusculoskeletal models to predict post-surgery walking function from pre-surgery walking data and the planned surgical decisions should explore using the Fixed or Shifted Synergy Control method to model the participant’s post-surgery neural control strategy.

Compared to previous studies, the present study collected more extensive walking data, with the data being available both before and after surgery. In part because custom prosthesis reconstruction has become a viable option only recently, few studies have performed instrumented gait analyses of this patient population (Akiyama et al., 2016; Wingrave and Jarvis, 2019; Valente et al., 2022). As the emphasis of these previous studies was on evaluating post-surgery walking function, pre-surgery walking data were not available from the same patients. No previous study reported ground reaction data for all three force components or joint moment data for all lower body joints. Only one study reported EMG data collected from six muscles in the operated leg. Thus, while limited to a single participant, the experimental walking data collected for the present study will be a valuable resource that other researchers can use to perform their own investigations of how internal hemipelvectomy surgery with custom prosthesis reconstruction affects post-surgery walking function.

While the extensive walking data collected for this study allowed for a detailed analysis of walking function and neural control changes between pre- and post-surgery conditions after plateau in recovery, the ultimate goal for these data is to facilitate the development of personalized neuromusculoskeletal modeling methods that can predict a patients’s post-surgery walking function reliably given the patients’s pre-surgery walking data and the surgical decisions being planned by the orthopedic oncologist. Calibration of personalized neuromusculoskeletal models currently requires extensive walking data, including motion capture, ground reaction, and EMG data. Furthermore, these data must be available before and after surgery so that the ability of the patient’s pre-surgery model to predict post-surgery walking function can be evaluated. One of the key challenges in predicting post-surgery walking function is that the patient’s post-surgery neural control strategy must be predicted at the same time. Thus, the availability of extensive pre- and post-surgery walking data from the same participant provides a unique opportunity to develop a hypothesis for how to model a patient’s post-surgery neural control strategy starting from the patient’s pre-surgery walking data.

The fact that assumed neural control strategy (e.g., minimize sum of squares of muscle activations) significantly impacts predicted walking motion (Ackermann and van den Bogert, 2010; Meyer et al., 2016) begs the question of which neural control assumption should be used for generating predictive walking simulations for some new situation (e.g., following surgery). At a high-level, two different neural control assumptions representing two different philosophies have been used in published studies. The first neural control assumption, which is used in most published studies (Fox et al., 2009; Koelewijn and van den Bogert, 2016; Lin et al., 2018; Song and Geyer, 2018; Falisse et al., 2019; Pitto et al., 2019; Miller and Esposito, 2021; Santos et al., 2021; Cseke et al., 2022; Hu et al., 2022; Bianco et al., 2023), will be termed “absolute control.” Absolute control involves minimizing one or more quantities in an absolute sense (i.e., relative to zero), such as minimizing the sum of squares of predicted muscle activations regardless of the walking situation or treatment. The second neural control assumption, which has only been used in publications by the corresponding author’s research group (Meyer et al., 2016; Sauder et al., 2019; Vega et al., 2022), will be termed “relative control.” Relative control involves minimizing one or more quantities relative to some non-zero baseline condition, such as minimizing the sum of squares of differences between predicted post-surgery synergy activations and calculated pre-surgery synergy activations. To the corresponding author’s knowledge, Meyer et al. (2016) (which minimized changes in synergy activations away from a baseline walking condition) is the only predictive walking simulation published to date that has been validated experimentally using walking data collected from the same participant under some new condition.

Because relative control requires a baseline neural control strategy, it is consistent with the concepts of neural adaptation and motor exploration. Neural adaptation implies that a patient possesses a baseline neural control strategy and then the CNS moves the patient’s neural control strategy away from this baseline in response to some new situation (e.g., split-belt treadmill training - Reisman et al., 2009). Motor exploration says that the CNS finds a neural control strategy for some new situation (e.g., post-surgery) by searching locally in the neighborhood of a baseline neural control strategy (e.g., pre-surgery) (Uehara et al., 2019). In contrast, absolute control is inconsistent with neural adaptation and motor exploration, plus researchers have yet to identify the absolute quantities that the CNS is minimizing (or maximizing) to produce walking under a wide variety of conditions by individuals without and with neurological impairment. For example, even if minimizing the sum of squares of muscle activations were optimal for healthy individuals without neurological impairment, it is unlikely to be optimal for individuals with cerebral palsy or stroke who possess neurological impairment. Thus, by applying the concept of relative control to muscle synergies, one can transform the hard problem of predicting post-surgery muscle activations for a large number of muscles (as required to generate reliable post-surgery predictive walking simulations) into the easier problem of predicting post-surgery muscle synergy changes in a lower dimensional space.

Despite a post-surgery gait pattern that visually appeared to be only mildly abnormal, the participant’s gait pattern was substantially altered by the surgery. First and foremost, the participant decreased his self-selected walking speed from 1.0 m/s pre-surgery to 0.5 m/s post-surgery. Since self-selected walking speed was cut in half, it is not surprising that the participant exerted less propulsive and braking force following surgery (Figure 2). Second, the participant exhibited a shortened swing phase for the non-operated leg post-surgery, likely a compensatory mechanism to reduce operated leg single-limb support time. Third, the participant exhibited post-surgery compensatory gait changes consistent with a Trendelenburg-Duchenne gait pattern (Kiernan et al., 2018). These changes included pelvis drop to the non-operated side coupled with lumber bending to the operated side during operated side single leg support (Figure 4), which is consistent with impaired hip abductor function (Supplementary Table S1) and a significantly reduced hip abduction moment in the operated leg following surgery (Figure 5). Fourth, the participant also exhibited post-surgery compensatory gait changes consistent with stiff knee gait (Fujita et al., 2022). The participant exhibited a hyperextended knee from mid to late stance phase (Figure 3A), producing a knee flexion moment rather than the expected knee extension moment. This atypical knee moment counteracted the external moment caused by the ground reaction force vector passing in front of rather than behind the knee, which in turn was caused by significant forward trunk lean following surgery (Figure 4). Though knee hyperextension and greater anterior trunk lean have been observed in individuals with quadriceps muscle weakness (Siegel et al., 2007; Sato and Maitland, 2008), the participant’s vastii muscles were not touched by the surgery, while his rectus femoris muscle was detached at its origin but later re-attached. Thus, while quadriceps muscle weakness seems unlikely, the participant’s post-surgery EMG data suggests that quadriceps muscle activation impairment may have occurred (Supplementary Figure S3A) for reasons that remain unclear.

To support our ultimate goal of developing a computational methodology that can predict a patient’s post-surgery walking function from his pre-surgery walking data and the planned surgical decisions, we calculated muscle synergies that were not only electromyographically consistent but also kinetically consistent. In nearly all published studies, muscle synergies are calculated from experimental EMG data alone, making them only electromyographically consistent. If the calculated muscle synergies were input into a neuromusculoskeletal model of the participant, along with experimental joint kinematic data, the model would not produce the participant’s experimental joint moments and thus would be kinetically inconsistent. In contrast, muscle synergies in our study were calculated from experimental EMG, joint kinematic, and joint moment data via calibrated EMG-driven musculoskeletal models, making them not only electromyographically consistent but also kinetically consistent. Thus, our calculated muscle synergies should provide an excellent starting point for future predictive simulations of post-surgery walking function.

Two observations support the conclusion that the Fixed or Shifted Synergy Control method is a better choice than the Fixed Synergy Vector method for predicting post-surgery muscle controls within a predictive simulation of post-surgery walking function. First, the largest pre-to post-surgery changes in neural control, as quantified using muscle synergies, occurred in the participant’s synergy vectors rather than his synergy excitations or activations. The cosine similarity metric showed that there was higher similarity between pre- and post-surgery synergy controls than between pre- and post-surgery synergy vectors (Table 2). The fact that the participant’s synergy vectors exhibited substantial pre- to post-surgery changes for both legs may be the first published evidence that an individual with healthy neural control is able to change his coordination strategy in both legs in response to the loss of a significant number of muscles in one leg. Second, the Fixed and Shifted Synergy Control methods worked much better than did the Fixed Synergy Vector method for reconstructing post-surgery muscle controls using pre-surgery synergy information (Figure 8). The Fixed Synergy Vector method assumes that the coordination strategy for lower extremity muscles stays more or less the same follow surgery. However, this method was incapable of accurately reconstructing post-surgery muscle controls, whereas the Fixed and Shifted Synergy Control methods achieved highly accurate post-surgery reconstructions.

Another important question for predicting post-surgery changes in neural control is whether the neural control model should represent muscle excitations or muscle activations. When post-surgery muscle controls were reconstructed using the Fixed or Shifted Synergy Control method, the highest reconstruction accuracy for both legs was achieved when the reconstruction process was applied to muscle activations rather than muscle excitations (Figure 8). This difference may be related to the fact that muscle activations are “closer” to biomechanical function than are muscle excitations, which must be time delayed and passed through activation dynamics to be transformed into muscle activations. Electromechanical delay, which was roughly constant for all muscles, would equate to different percentages of the gait cycle for different walking speeds. The average calibrated electromechanical delay for all muscles was 83.7 milliseconds. This delay equated to an average 7.6% of the pre-surgery gait cycle (1.0 m/s) but only 5.6% of the post-surgery gait cycle (0.5 m/s). Thus, from the standpoint of a normalized gait cycle, one might expect pre- and post-surgery muscle activations to be better synchronized than pre- and post-surgery muscle excitations.

While reconstruction of post-surgery muscle controls using the Fixed or Shifted Synergy Control method generally improved with an increased number of muscle synergies, it is important to consider how many synergies should be used. Given our ultimate goal of generating predictive simulations of post-surgery walking function, the lowest number of synergies should be chosen that can reconstruct post-surgery muscle controls accurately. An accurate reconstruction capable of controlling a predictive walking simulation requires a VAF value higher than 95% overall and 85% for individual muscles (Meyer et al., 2016). For the Fixed Synergy Control method with five synergies, the mean overall VAF value for muscle activation reconstruction was 95.2% for the non-operated leg and 95.6% for the operated leg, though overall VAF values for several gait cycles failed to reach the 95% threshold (Figure 8). Using six synergies, the overall and individual muscle VAF values for reconstructed muscle activations both exceeded the previous requirements. When seven synergies were used, the VAF improvements started to diminish, especially for the operated leg (*p* = 0.1065). For the Shifted Synergy Control method, only five synergies were needed to achieve the desired overall and individual muscle VAF criteria for both the non-operated and operated leg, representing a less expensive computational option for predictive simulations. Thus, for future predictive simulation studies of post-surgery walking function, it would be difficult to justify using more than five or six synergies per leg.

In addition to quantifying changes in neural control, quantifying changes in muscle-tendon model parameters values is helpful for future studies that perform predictive simulations of post-surgery walking function. These parameter values, namely, optimal muscle fiber length and tendon slack length, are crucial for modeling the force-generating characteristics of the patient’s lower extremity muscles. Based on a comparison of the participant’s pre- and post-surgery calibrated neuromusculoskeletal models, changes in these parameter values were generally small apart from some outliers (Figure 6). These outliers generally occurred for two types of muscles. The first type included muscles without experimental EMG data (Table 1). For example, flexor hallucis longus (FHL) in the non-operated leg had its optimal muscle fiber length increased by 0.045 m or 132%. Since the excitation of such muscles had to be estimated using synergy extrapolation, associated muscle-tendon model parameter values tended to be less accurate than for muscles with experimental EMG data. The second type included muscles that were somehow affected by the surgery. For example, vastus lateralis (VL) in the operated leg had its optimal fiber length increase by 0.070 m or 40%. As noted previously, the participant avoided using this muscle following surgery for reasons that remain unclear. Parameter tuning for such muscles during model calibration was possibly more aggressive to overcome the deficits in activation. When outlier muscles were excluded, changes in these model parameter values were much smaller. The median change in optimal muscle fiber length was 0.013 and −0.0005 m for the operated and non-operated leg, respectively. The median change in tendon slack length was −0.008 and −0.005 m for the two legs. The median values of percent change were also small (13%, −1%, −3%, and −5%, respectively). Thus, for future studies that seek to predict post-surgery walking function starting from a pre-surgery walking model, pre-surgery muscle-tendon model parameter values should provide a reasonable approximation.

This study possesses several limitations related to data generalizability and computational methodology. First, this study analyzed data collected from a single pelvic sarcoma patient. Given the significant heterogeneity between patients with this pathology, it is unknown whether the conclusions drawn from the present study can be generalized to other patients with a pelvic sarcoma. More data collected from more patients are needed to determine generalizability of these results. The number of patients with a pelvic sarcoma is limited, plus it is challenging to find patients who are willing to come in for gait testing prior to surgery and after plateau in recovery. However, the findings of this study can still be used as the foundation for the first predictive simulations of post-surgery walking for this patient population. Second, during calibration of post-surgery EMG-driven models, the peak isometric force values of the remaining hip muscles in the operated leg were set at 100% of their pre-surgery values and were not modified despite the possibility that some muscles may have been weakened by the surgery. Use of pre-surgery peak isometric force values could lead to underestimation of activations for certain hip muscles, especially those that were detached and later reattached. Third, although measured excitations were available for the majority of lower extremity muscles (Table 1), excitations of multiple unmeasured muscles still needed to be estimated using synergy extrapolation (Bianco et al., 2018; Ao et al., 2020; Ao et al., 2022). While this method of estimating unmeasured muscle excitations has been shown to be reliable for a small number of muscles, its effectiveness when extended to multiple muscles has yet to be verified. Fourth, the methods investigated for reconstructing post-surgery muscle controls from pre-surgery muscle synergy information were evaluated using only experimental muscle excitations and activations. The reliability of these methods for predicting the activations of muscles without experimental EMG data is unknown.

In conclusion, this study performed extensive analyses of walking function and neural control changes for a single pelvic sarcoma patient following internal hemipelvectomy surgery with custom pelvic prosthesis reconstruction. The participant exhibited substantial changes in his post-surgery walking function when quantified using experimental data, despite only minor abnormalities being observed visually. The observed changes could potentially provide valuable information for designing a personalized rehabilitation protocol for this participant. The participant also exhibited substantial changes in the coordination of his lower extremity muscles in both legs, as evidenced by pre-to post-surgery changes in his synergy vectors. Consequently, when pre-surgery muscle synergy information was used to reconstruct post-surgery muscle activations, the Fixed and Shifted Synergy Control methods that used pre-surgery synergy activations but found new synergy vectors produced the most accurate reconstructions (>95% VAF) and required only five or six synergies. Consequently, we recommend that future computational studies that seek to predict post-surgery walking function for this patient population start by using the patient’s pre-surgery synergy activations, thereby greatly simplifying the process of predicting the patient’s post-surgery neural control strategy.

## Data Availability

The experimental data and computational neuromusculoskeletal models used to perform this study are available at https://simtk.org/projects/pelvic_sarcoma.
