# Supplementary material for: Changes in walking function and neural control following pelvic cancer surgery with reconstruction
Source: Front Bioeng Biotechnol. 2024 May 17;12:1389031. doi: 10.3389/fbioe.2024.1389031 (PMC11140731; doi:10.3389/fbioe.2024.1389031)
Supplement: Supplementary file 1 [file DataSheet1.pdf]

Supplementary Material

Changes in Walking Function and Neural Control following Pelvic Cancer Surgery with Reconstruction

Geng Li[1], Di Ao[1], Marleny M. Vega[1], Payam Zandiyeh[2], Shuo-Hsiu Chang[3], Alexander N. Penny[4], Valerae O. Lewis[4] and Benjamin J. Fregly[1*]

[1]Rice Computational Neuromechanics Laboratory, Department of Mechanical Engineering, Rice University, Houston, TX, USA

[2]Biomotion Laboratory, Department of Orthopedic Surgery, McGovern Medical School at the University of Texas Health Science Center at Houston, Houston, TX, Country

[3]Department of Physical Medicine and Rehabilitation, McGovern Medical School at the University of Texas Health Science Center at Houston, Houston, TX, Country

[4]Department of Orthopedic Oncology, University of Texas MD Anderson Cancer Center, Houston, TX, Country

*** Correspondence:** Benjamin J. Fregly (fregly@rice.edu)

1 Supplementary Figures and Tables

1.1 Supplementary Tables

Supplementary Table S1. Status of the muscles on the operated hemipelvis (right) following an internal hemipelvectomy surgery.

<table>
<tr><th>Body Segment</th><th>Muscle Names (Abbreviation)</th><th>Muscle Groups</th><th>Surgical Decisions</th></tr>
<tr><td rowspan="24">Leg</td><td>adductor brevis (ADB)</td><td>adductor brevis</td><td>Removed</td></tr>
<tr><td>adductor longus (ADL)</td><td>adductor longus</td><td>Removed</td></tr>
<tr><td>adductor magnus distal (ADM1)
adductor magnus ischial (ADM2)
adductor magnus middle (ADM3)
adductor magnus proximal (ADM4)</td><td>adductor magnus</td><td>Removed</td></tr>
<tr><td>biceps femoris long head (BFLH)
biceps femoris short head (BFSH)</td><td>biceps femoris</td><td>Reattached</td></tr>
<tr><td>gemellus (GEM)</td><td>gemellus</td><td>Removed</td></tr>
<tr><td rowspan="2">gluteus maximus superior (GMA1)
gluteus maximus middle (GMA2)
gluteus maximus inferior (GMA3)</td><td rowspan="2">gluteus maximus</td><td>Reattached</td></tr>
<tr><td>Removed</td></tr>
<tr><td>gluteus medius anterior (GME1)
gluteus medius middle (GME2)
gluteus medius posterior (GME3)</td><td>gluteus medius</td><td>Reattached</td></tr>
<tr><td>gluteus minimus anterior (GMI1)
gluteus minimus middle (GMI2)
gluteus minimus posterior (GMI3)</td><td>gluteus minimus</td><td>Removed</td></tr>
<tr><td>gracilis (GRAC)</td><td>gracilis</td><td>Removed</td></tr>
<tr><td>iliacus (IL)</td><td>iliacus</td><td>Removed</td></tr>
<tr><td>pectineus (PECT)</td><td>pectineus</td><td>Reattached</td></tr>
<tr><td>piriformis (PIRI)</td><td>piriformis</td><td>Removed</td></tr>
<tr><td>psoas major superior (PS1)
psoas major middle (PS2)
psoas major inferior (PS3)</td><td>psoas</td><td>Kept</td></tr>
<tr><td>quadratus femoris (QF)</td><td>quadratus femoris</td><td>Removed</td></tr>
<tr><td>rectus femoris (RF)</td><td>rectus femoris</td><td>Reattached</td></tr>
<tr><td>sartorius (SART)</td><td>sartorius</td><td>Reattached</td></tr>
<tr><td>semimembranosus (SM)</td><td>semimembranosus</td><td>Reattached</td></tr>
<tr><td>semitendinosus (ST)</td><td>semitendinosus</td><td>Reattached</td></tr>
<tr><td>tensor fasciae latae (TFL)</td><td>tensor fasciae latae</td><td>Reattached</td></tr>
<tr><td>vastus medialis (VM)</td><td>vastus medialis</td><td>Kept</td></tr>
<tr><td>vastus intermedius (VI)</td><td>vastus intermedius</td><td>Kept</td></tr>
<tr><td>vastus lateralis (VL)</td><td>vastus lateralis</td><td>Kept</td></tr>
<tr><td rowspan="6">Trunk</td><td>internal obliques (IO)</td><td>internal obliques</td><td>Reattached</td></tr>
<tr><td>external obliques (EO)</td><td>external obliques</td><td>Reattached</td></tr>
<tr><td>erector spinae (ES)</td><td>erector spinae</td><td>Removed</td></tr>
<tr><td>multifidus (MF)</td><td>multifidus</td><td>Kept</td></tr>
<tr><td>quadratus lumborum (QL)</td><td>quadratus lumborum</td><td>Removed</td></tr>
<tr><td>rectus abdominis (RA)</td><td>rectus abdominis</td><td>Reattached</td></tr>
</table>

Supplementary Table S2. Mean absolute errors in the joint moments estimated by EMG-driven models. HipFE – hip flexion/extension, HipAA – hip adduction/abduction, HipRot – hip internal/external rotation, KneeFE – knee flexion/extension, AnkleDP – ankle dorsi/plantar flexion, Subtalar IE – subtalar inversion/eversion.

		Mean Absolute Error in Joint Moment Estimates (Nm)					
	Leg	HipFE	HipAA	HipRot	KneeFE	AnkleDP	SubtalarIE
Pre-surgery	R	3.49	5.01	2.08	4.23	4.36	1.50
	L	3.70	4.09	1.77	2.93	4.96	2.63
Post-surgery	R	2.95	2.54	1.64	4.09	2.94	2.84
	L	3.23	3.94	2.31	3.60	3.57	1.37

Supplementary Table S3. Shift required to achieve maximum cosine similarity between pre- and post-surgery synergy activations, for number of synergies n = 4, 5, 6, and 7. See Supplementary Figure 5 for the synergy activation curves.

	Operated Leg			
	n = 4	n = 5	n = 6	n = 7
Syn #1	-23	-17	-14	-2
Syn #2	-11	-10	-6	1
Syn #3	-3	-1	-1	5
Syn #4	-10	-4	2	8
Syn #5		-2	3	3
Syn #6			2	2
Syn #7				4
Mean absolute	11.8	6.8	4.7	3.6
	Non-operated Leg			
	n = 4	n = 5	n = 6	n = 7
Syn #1	-5	-9	-5	-6
Syn #2	0	-5	-2	-4
Syn #3	14	1	4	0
Syn #4	14	7	1	1
Syn #5		5	0	0
Syn #6			-8	-1
Syn #7				-8
Mean absolute	8.3	5.4	3.3	2.9

Supplementary Table S4. Bonferroni correction to the significance level used by the statistical tests for various cases and scenarios of samples

Each sample consists of 10 VAF values, each for the reconstructed muscle control of one post-surgery gait cycle, using a specific combination of methodological choices (e.g., Fixed Synergy Vector method applied to muscle excitations using 4 synergies).

$$p = \frac{0.05}{N}$$

		Reconstruction Option	Muscle Control Types	Number of Synergies	Leg	Number of Tests (N)	p-value after correction
**Case 1**	**Scenario 1**	Fixed Synergy Vector versus Fixed Synergy Control	Excitation Activation	4,5,6,7	L, R	16	0.003
	**Scenario 2**	Fixed Synergy Vector versus Shifted Synergy Control	Excitation Activation	4,5,6,7	L, R	16	0.003
	**Scenario 3**	Fixed Synergy Control versus Shifted Synergy Control	Activation	4,5,6,7	L, R	8	0.006
**Case 2**	**Scenario 1**	Fixed Synergy Control	Excitation versus Activation	4,5,6,7	L, R	8	0.006
**Case 3**	**Scenario 1**	Fixed Synergy Control Shifted Synergy Control	Activation	4 versus 5	L, R	4	0.0125
	**Scenario 2**	Fixed Synergy Control Shifted Synergy Control	Activation	5 versus 6	L, R	4	0.0125
	**Scenario 3**	Fixed Synergy Control Shifted Synergy Control	Activation	6 versus 7	L, R	4	0.0125

1.2 Supplementary Figures

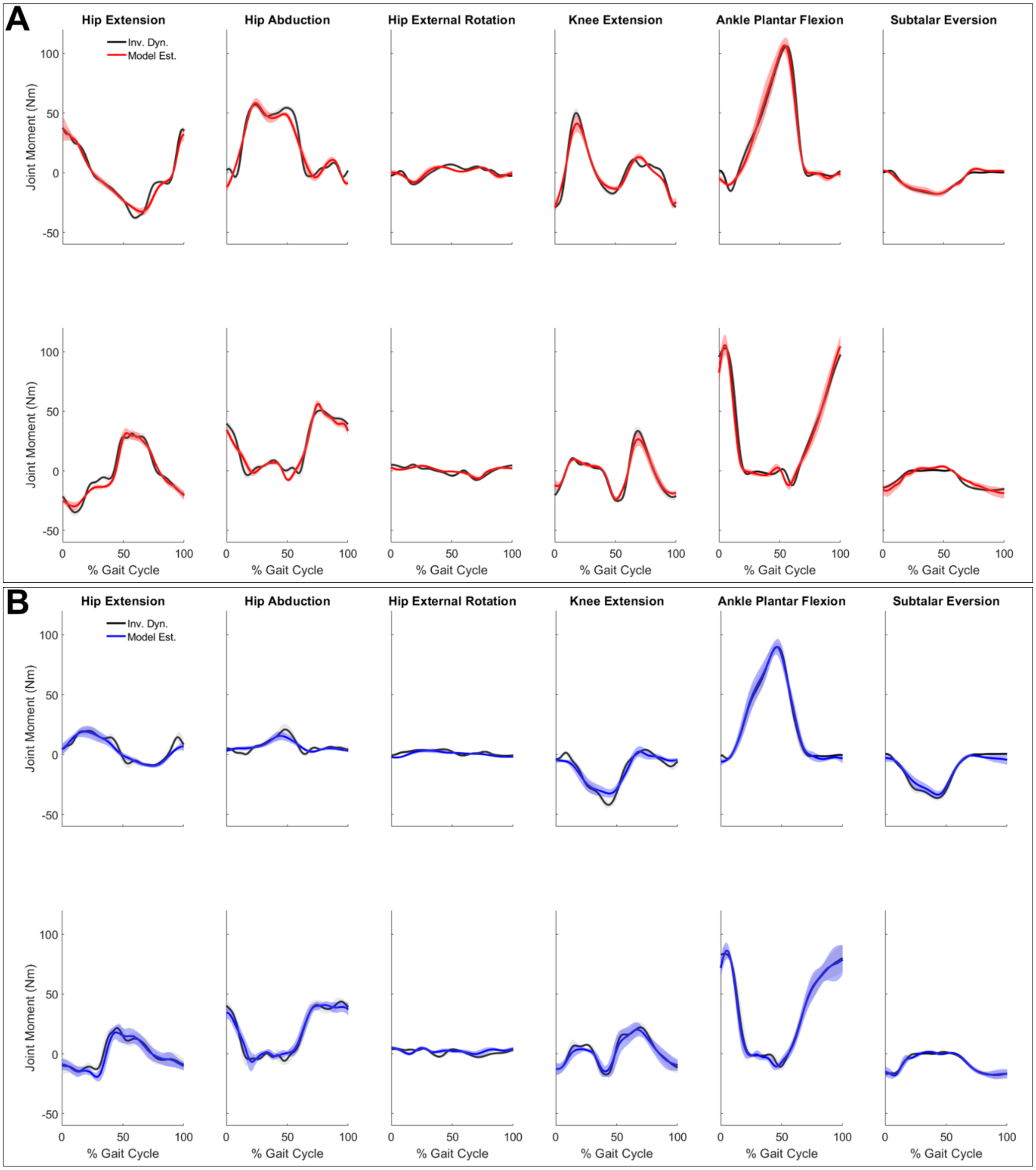

Supplementary Figure S1. Lower extremity joint moments estimated by the EMG-driven models and calculated from inverse dynamics for **A.** Pre-surgery joint moments: model-estimated (red), inverse dynamics (dark). **B.** Post-surgery joint moments: model-estimated (blue), inverse dynamics (dark). The solid curves represent mean and the shaded areas represent ± 1 standard deviation of joint moments.

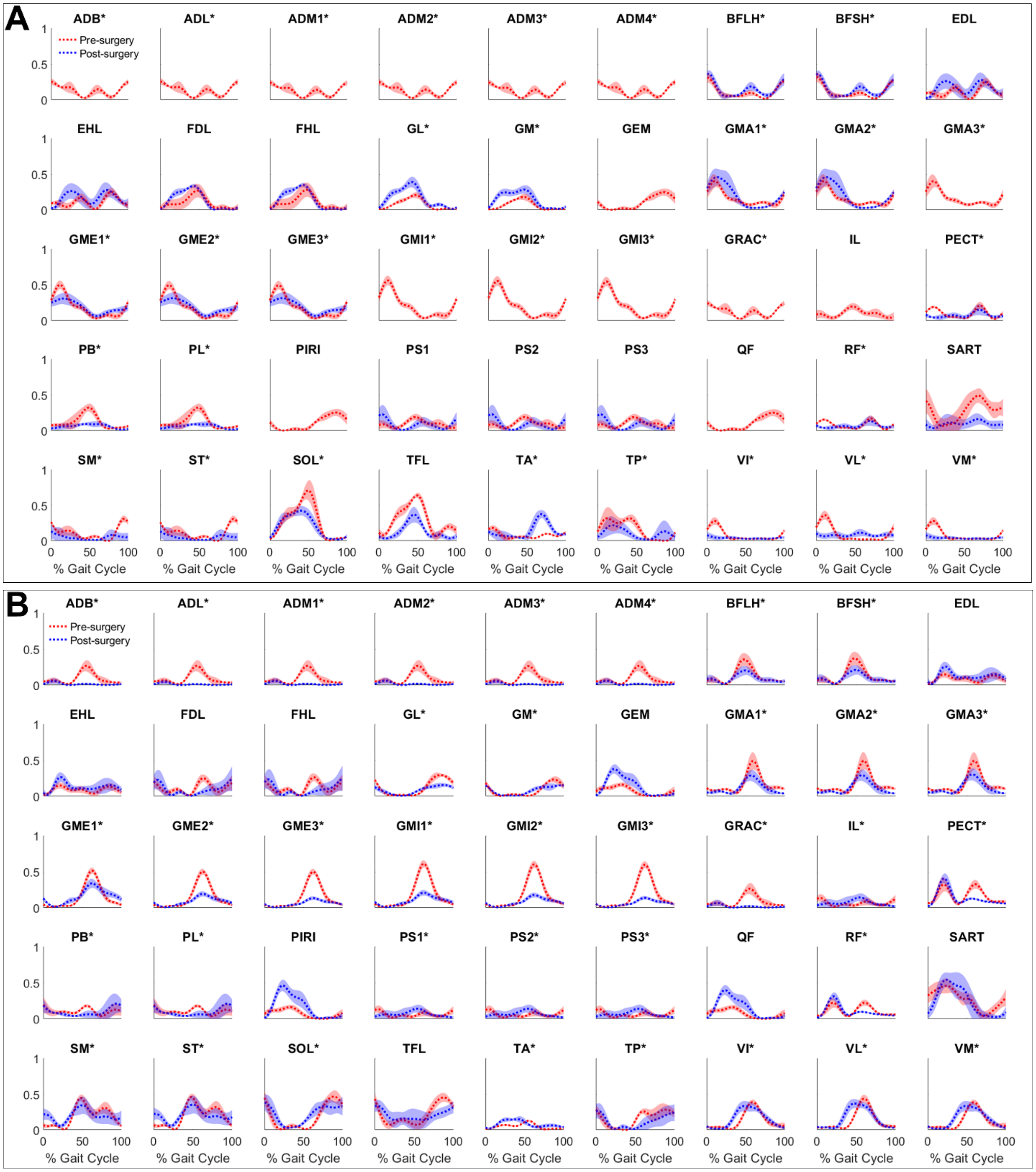

Supplementary Figure S2. Excitations (mean ± standard deviation across 10 gait cycles) of lower extremity muscles pre-surgery (red) and post-surgery (blue) for **A.** operated leg and **B.** non-operated leg. * indicates EMG data of the muscles were collected during gait trials.

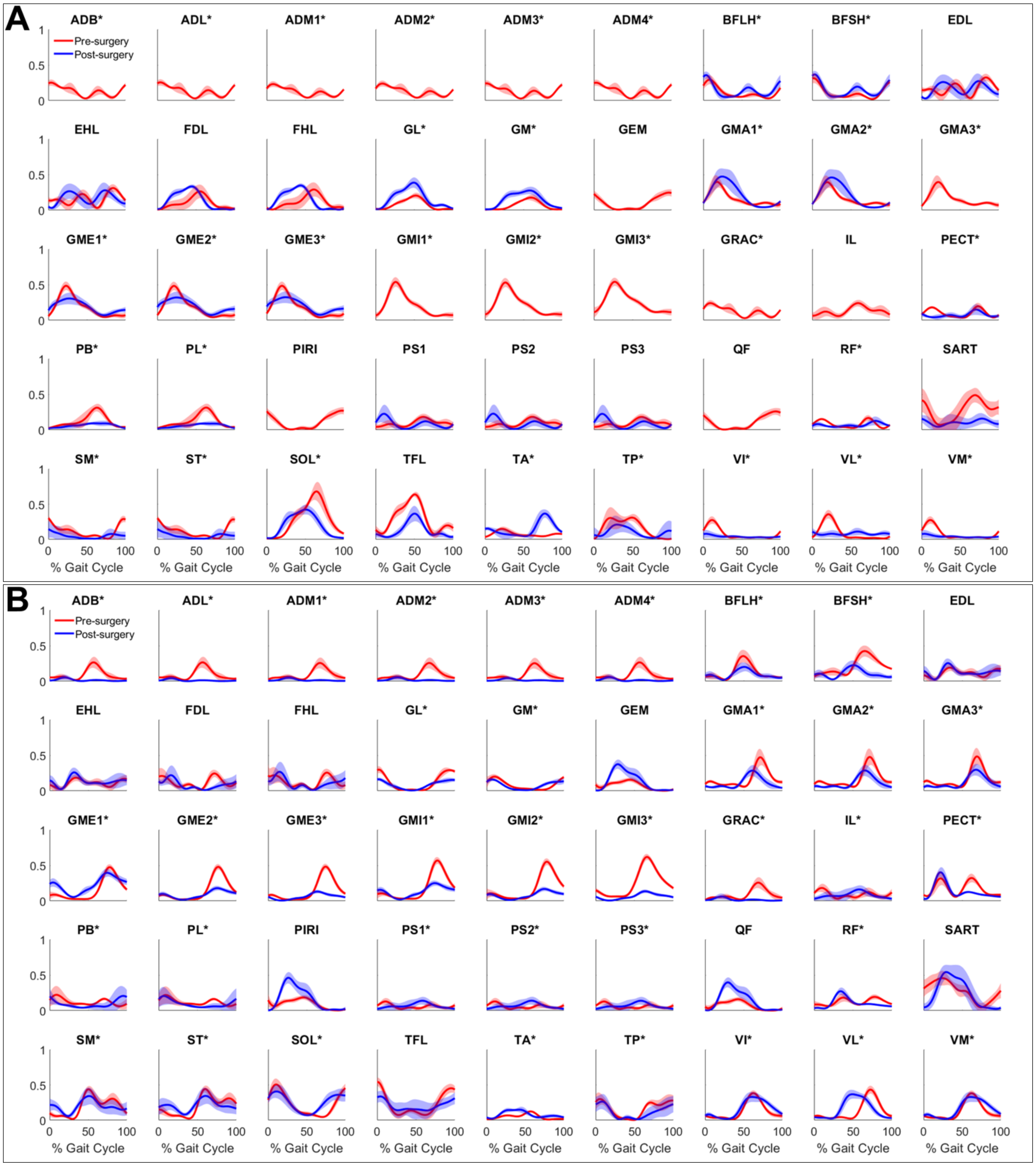

Supplementary Figure S3. Activations (mean ± standard deviation across 10 gait cycles) of lower extremity muscles pre-surgery (red) and post-surgery (blue) for **A.** operated leg and **B.** non-operated leg. * indicates EMG data of the muscle were collected during gait trials.

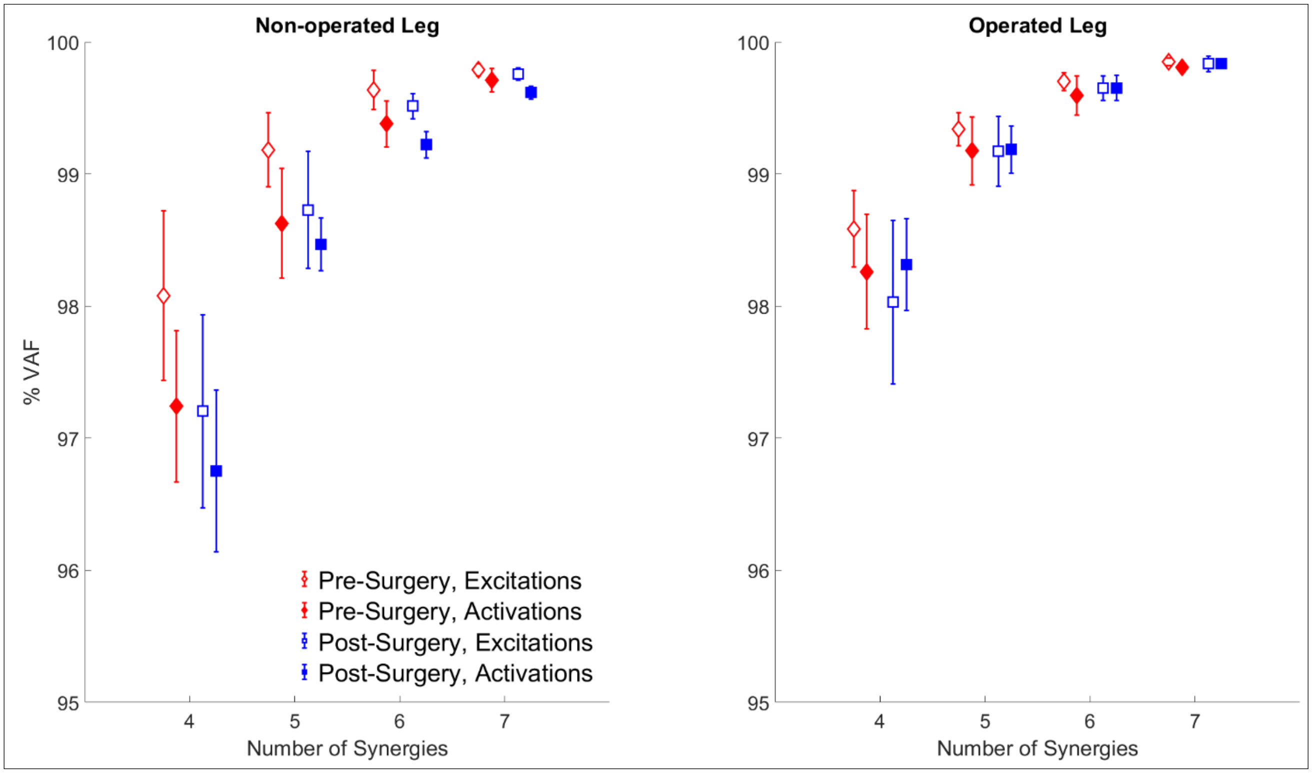

Supplementary Figure S4. Variability account for, or VAF (mean ± standard deviation across 10 gait cycles) of muscle excitations (empty markers) and activations (filled markers) by the muscle synergies for pre-surgery (red or diamond) and post-surgery (blue or square).

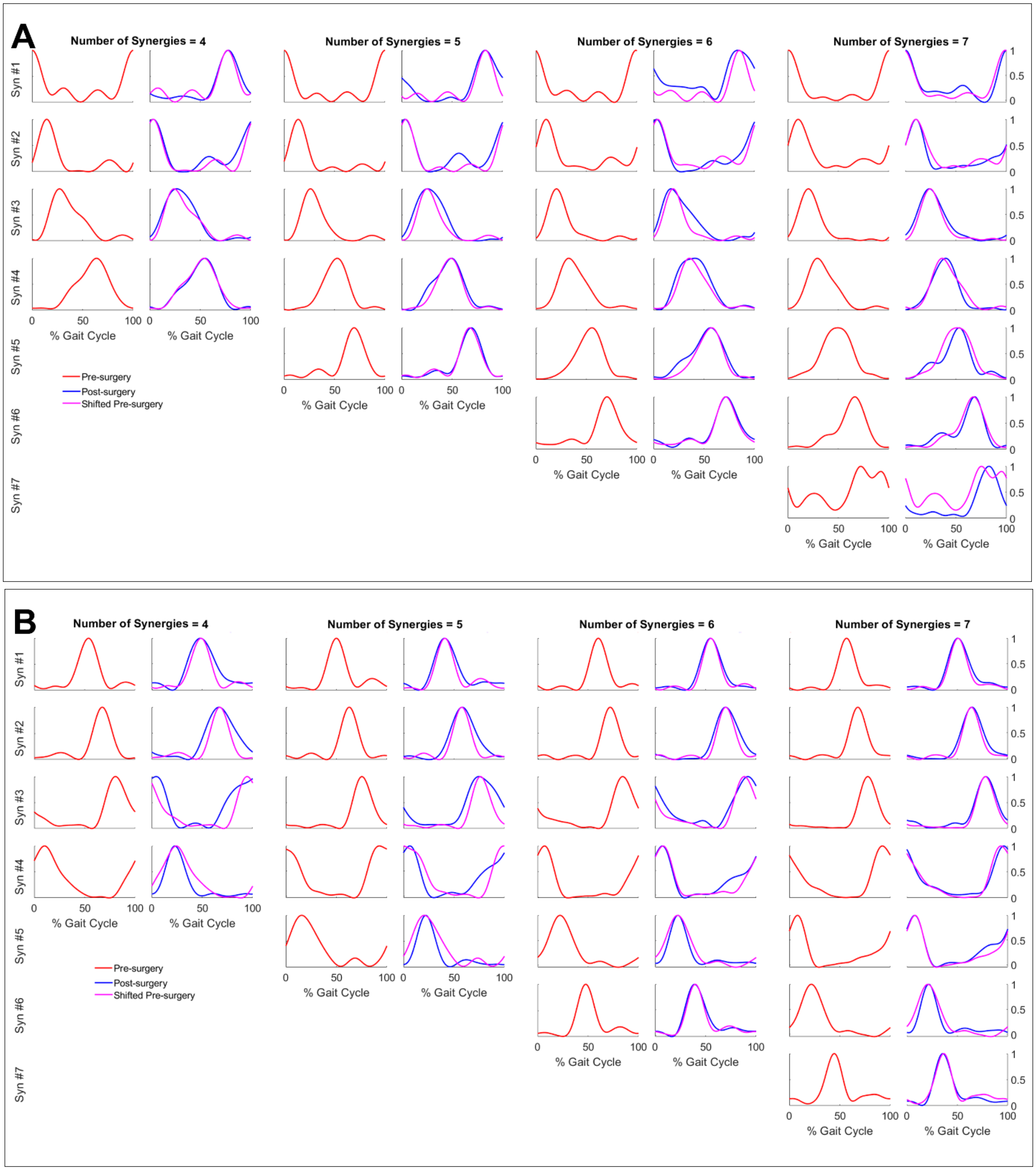

Supplementary Figure S5. Pre-surgery (red) and post-surgery (blue) synergy activations (mean across 10 gait cycles), as well as the shifted pre-surgery synergy activations (magenta) that had maximum cosine similarity with post-synergy activations for **A.** operated leg and **B.** non-operated leg. See Supplementary Table X for the shifts required to achieve maximum cosine similarity.

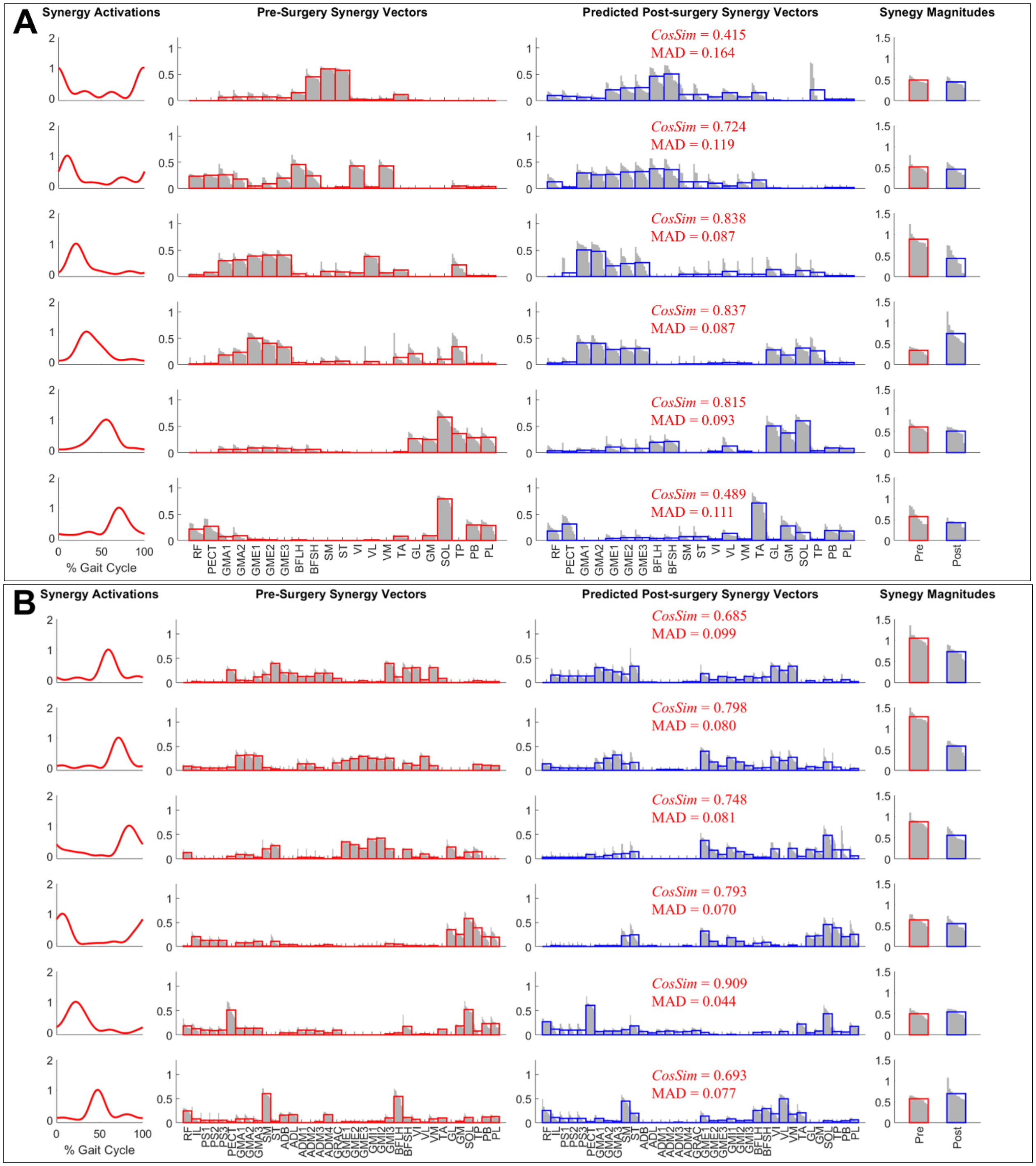

Supplementary Figure S6. Pre-surgery synergy commands and synergy vectors (red), and synergy vectors required to reconstruct post-surgery muscle activations using Fixed Synergy Control method (blue) for **A.** operated leg and **B.** non-operated leg. The red and blue bars represent the mean values of cycle-specific synergy vector weights (grey). Cosine similarity and mean absolute difference between the two set of synergy vectors were calculated using the mean values (red and blue bars).
